# Relationships of N6-Methyladenosine-Related Long Non-Coding RNAs With Tumor Immune Microenvironment and Clinical Prognosis in Lung Adenocarcinoma

**DOI:** 10.3389/fgene.2021.714697

**Published:** 2021-10-20

**Authors:** Jianhui Zhao, Xi Lin, Jinman Zhuang, Fei He

**Affiliations:** ^1^ Department of Epidemiology, School of Public Health, Southern Medical University, Guangzhou, China; ^2^ Department of Toxicology, School of Public Health, Southern Medical University, Guangzhou, China; ^3^ Department of Epidemiology and Health Statistics, School of Public Health, Fujian Medical University, Fuzhou, China; ^4^ Fujian Provincial Key Laboratory of Tumor Microbiology, Fujian Digital Tumor Data Research Center, Fujian Medical University, Fuzhou, China

**Keywords:** lung adenocarcinoma, N6-methylandenosine, long non-coding RNA, prognosis, tumor immune microenvironment

## Abstract

**Background:** Lung adenocarcinoma (LUAD) is the major subtype of lung cancer and is associated with very high mortality. Emerging studies have shown that N6-methyladenosine (m6A)-related long non-coding (lnc) RNAs play crucial roles in tumor prognosis and the tumor immune microenvironment (TME). We aimed to explore the expression patterns of different m6A-related lncRNAs concerning patient prognosis and construct an m6A-related lncRNA prognostic model for LUAD.

**Methods:** The prognostic value of m6A-related lncRNAs was investigated in LUAD samples from The Cancer Genome Atlas (TCGA). Potential prognostic m6A-related lncRNAs were selected by Pearson’s correlation and univariate Cox regression analysis. Patients were divided into clusters using principal component analysis and the m6A-related lncRNA prognostic signature was calculated using least absolute shrinkage and selection operator (LASSO) Cox regression analysis.

**Results:** Based on 91 prognostic m6A-related lncRNAs, we identified two m6A-related-lncRNA pattern clusters with different overall survival (OS) and different TMEs. We subsequently verified our findings multidimensionally by constructing a 13 m6A-related lncRNA prognostic signature (m6A-LPS) to calculate the risk score, which was robust in different subgroups. The receiver operating characteristic (ROC) curves and concordance index demonstrated that m6A-LPS harbored a promising ability to predict OS in TCGA data set and independent GSE11969 cohort. The risk score was also related to OS, TME, and clinical stage, and the risk score calculated by our model was also identified as independent prognostic predictive factors for LUAD patients after adjustment for age, smoking, gender, and stage. Enrichment analysis indicated that malignancy and drug resistance-associated pathways were more common in cluster2 (LUAD-unfavorable m6A-LPS). Furthermore, the results indicated that the signaling pathway enriched by the target gene of 13 m6A-related lncRNAs may be associated with metastasis and progression of cancer according to current studies.

**Conclusion:** The current results indicated that different m6A-related-lncRNA patterns could affect OS and TME in patients with LUAD, and the prognostic signature based on 13 m6A-related lncRNAs may help to predict the prognosis in LUAD patients.

## Introduction

Lung cancer is associated with very high mortality worldwide, compared with breast, prostate, and pancreatic cancers (Siegel et al., 2020). Approximately 85% of lung cancer cases are non-small-cell lung cancer (NSCLC) (Ma et al., 2013), with lung adenocarcinoma (LUAD) accounting for approximately 47–82% of all NSCLC cases (Fitzmaurice et al., 2017; Siegel et al., 2020). Despite recent developments in the screening, diagnosis, and precise treatment of LUAD, its 5 years survival rate is only about 15% because many patients are diagnosed with advanced disease (Denisenko et al., 2018). There is thus a need to explore novel diagnostic, therapeutic, and prognostic targets for LUAD.

N6-methyladenosine (m6A) modification has been identified as an important epigenetic methylated modification of long non-coding RNAs (lncRNAs), with abundant biological functions (Zhao et al., 2017). Modification of m6A is regulated by methyltransferases, signal transducers, and demethylases, and is involved in RNA epigenetic processes (Zaccara et al., 2019). Recent research has focused on the role of m6A modification in regulating carcinogenesis and cancer progression, including in LUAD (Li et al., 2020; Zhang et al., 2020; Wang et al., 2021a; Wu et al., 2021). For instance, METTL3-mediated m6A mRNA modification of FBXW7 suppressed LUAD (Wu et al., 2021), while FTO and YTHDF1 affected bladder cancer (Tao et al., 2021) and ovarian cancer progression (Liu et al., 2020), and YTHDC2 inhibits LUAD tumorigenesis by suppressing SLC7A11-dependent antioxidant function (Ma et al., 2021). As suggested by a growing body of evidence, dysregulation of RNA modifications may be a key regulatory mechanism in a variety of diseases, and RMVar and RMdisease were developed to present the RNA modifications on potential disease association (Chen et al., 2021; Luo et al., 2021).

Tumor malignancy was shown to be regulated by aberrant lncRNA, and the dysregulation of lncRNAs, including immune-related (Wang et al., 2021b; You et al., 2021), autophagy-related (Jiang et al., 2021) and T cell-related (Duan et al., 2020) lncRNAs had critical effects on the progression and prognosis of LUAD (Zhou et al., 2018; Xu et al., 2019; Zhou et al., 2019). However, the specific interactions between m6A regulators and lncRNAs in cancer remain unclear, and studies investigating the effects of m6A modification on lncRNA-dependent LUAD, prognosis, and tumor microenvironment are currently lacking. Exploring m6A-related modifications of lncRNAs involved in LUAD progression may thus aid the identification of biomarkers that can act as useful diagnostic, therapeutic, and prognostic targets.

In this study, we used bioinformatics and statistical analyses to determine the prognostic significance of m6A-related lncRNAs in LUAD patients (*n* = 486) based on The Cancer Genome Atlas (TCGA) dataset. We identified 91 m6A-related lncRNAs with potential prognostic value in LUAD patients. We then established a 13 m6A-related lncRNA prognostic signature model (m6A-LPS) to predict the survival time and morbid status of LUAD patients. The prognosis and tumor immune microenvironment (TME) differed between patients in the low- and high-risk subgroups. These results suggested that underlying m6A-related lncRNAs may provide useful prognostic and diagnostic biomarkers, which may help to improve personalized therapies in patients with LUAD.

## Materials and Methods

### Source of Datasets and m6A-Related Genes

We acquired RNA transcriptome data as Fragments Per Kilobase per Million (FPKM), and clinical information for patients with LUAD were downloaded from TCGA-LUAD using the Genomic Data Commons (https://portal.gdc.cancer.gov/). We further analyzed the data for 551 samples, 497 tumors and 54 normal samples. We merged the clinical data for survival time, survival status, age, and sex using the Practical Extraction and Report Language (Perl). After reading and summarizing the published literature, we focused on 23 m6A-related genes with major roles in writing (*METTL3*, *METTL14*, *METTL16*, *WTAP*, *VIRMA* [*KIA1499*], *ZC3H13*, *RBM15*, and *RBM15B*), erasing (*FTO* and *ALKBH5*), and reading (*YTHDC1*, *YTHDC2*, *YTHDF1*, *YTHDF2*, *YTHDF3*, *HNRNPC*, *FMR1*, *LRPPRC*, *HNRNPA2B1*, *IGFBP1*, *IGFBP2*, *IGFBP3*, and *RBMX*). We extracted the matrix data for these 23 m6A-related genes from TCGA databases. In addition, the non-smoker was defined as a person who was not smoking at the time of the interview and has smoked less than 100 cigarettes in their life, and the smoker was defined as a person who has smoked at least 100 cigarettes in their life.

### Identification of lncRNAs Related to m6A in LUAD Patients

**FIGURE 1:**
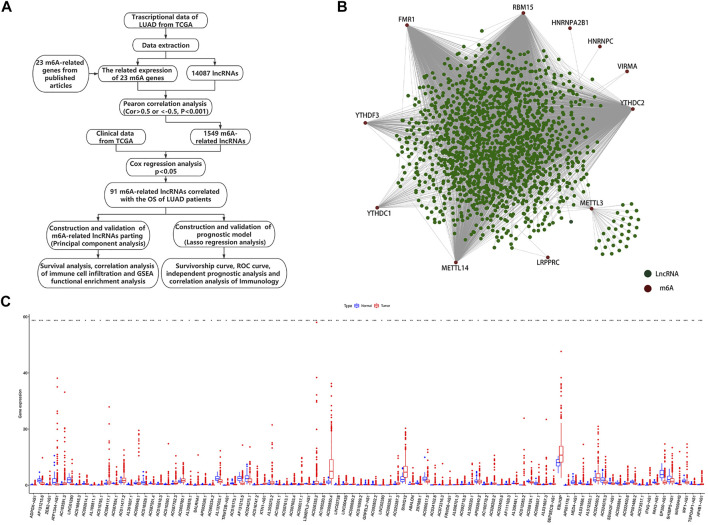
Identification of m6A-related lncRNAs in LUAD patients. **(A)** Study flow chart. **(B)** Network of correlations between m6A-related genes and m6A-related lncRNAs. **(C)** Expression chamber diagram of 91 prognostic m6A-related lncRNAs (*p < 0.05; **p < 0.01; ***p < 0.001).

We obtained detailed clinical information for 486 samples from the extracted clinical profiles and evaluated the survival outcomes of the LUAD patients based on overall survival (OS). We identified potential m6A-related lncRNAs based on a correlation coefficient R > |0.5| (*p*-value < 0.001), according to Pearson’s correlation analysis. We visualized the co-expression network of m6A-related genes and m6A-related lncRNAs using Cytoscape (version 3.8.0). Potential m6A-related lncRNAs associated with survival were identified by univariate Cox regression analysis using data that matched the patient ID numbers with the expression levels of m6A-related lncRNAs and survival outcomes. Figure 1A shows a detailed flow chart of the study. We analyzed the different expressions of m6A-related lncRNAs in the standard comparison model using the empirical Bayesian approach with the “limma” R package. The TCGA cohort was clustered into different groups by consensus expression of prognostic m6A-related lncRNAs with the “ConsensusClusterPlus” package (Wilkerson and Hayes, 2010).

### Survival and Pathway Analyses Between Clusters 1 and 2

We compared survival outcomes between the two clusters using the “survival” and “survminer” R package. Differences in pathways between the two clusters were analyzed by Kyoto Encyclopedia of Genes and Genomes (KEGG) pathway analysis, with screening criteria of *p* < 0.05, minimum count of 5, and enrichment factor >0.15.

### Comparison of TME Between the Two Clusters

The TMEs for the two clusters were evaluated based on immune, stromal, and estimate scores calculated using the Estimation of STromal and Immune cells in MAlignant Tumour tissues using Expression data (ESTIMATE) algorithm (Yoshihara et al., 2013). Differences in 22 immune cell subtypes between the two clusters were identified using the “CIBERSORT” package, and levels of immune cells were compared.

### Construction of m6A-Related lncRNA Prognostic Model

The “caret” R package was used to divide the TCGA set (*n* = 468). Ultimately, 236 and 232 patients are included in train (construction of model) and test sets (validation of model), respectively. We constructed a 13 m6A-related lncRNA prognostic model (m6A-LPS) by Least Absolute Shrinkage and Selection Operator (LASSO) Cox regression analysis using the “glmnet” software package of R. This prognostic model was then used to assess the relationships between m6A-related lncRNAs and LUAD risk, according to the following formula:

$$\mathrm{Risk}\;\mathrm{score}=\sum_{i=1}^{n}Coefi\;\text{*}\;xi.$$

The clinical information and platform annotation information of the externally validation dataset were obtained from the GSE11969 data set of the Gene Expression Omnibus (GEO) database (http://www.ncbi.nlm.nih.gov/geo). A total of 149 LUAD patients were included in the validation dataset of this study.

### Analysis of Online Databases

The expression of prognostic m6A-related lncRNAs and m6A regulators were verified by Starbase (http://starbase.sysu.edu.cn/) and Gene Expression Profiling Interactive Analysis (GEPIA) (http://gepia.cancer-pku.cn/) online databases. The Starbase and Lnc2Cancer 3.0 (http://bio-bigdata.hrbmu.edu.cn/lnc2cancer/) databases were used to predict the target genes of 13 m6A-related lncRNAs. Further, the Kyoto Encyclopedia of Genes and Genomes (KEGG) was used to analyze the target genes.

### The Prediction of the Potential Target Genes of LncRNAs and the Analysis of Pathway Enrichment

We examined the correlation between the expression level of 13 m6A-related prognostic lncRNAs and each protein-coding gene (PCGs) using Pearson correlation coefficients. A total of 5,118 PCGs were expressed as highly correlated with at least one of 13 m6A-related prognostic lncRNAs (Pearson correlation coefficient >0.4 and *p* < 0.01). Further, we performed KEGG pathway analysis by applying the “clusterProfiler” R package with a threshold *p*-value < 0.05.

### The Identification of Potential m6A Modification Sites on 13 m6A-Related lncRNAs

The sequence-based RNA adenosine methylation site predictor (SRAMP) was used to identify potential m6A modification sites on all 13 m6A-related lncRNAs (Zhou et al., 2016).

### Statistical Analysis

All statistical analyses were performed using R software (version 3.6.1, http://www.R-project.org). Survival outcomes for the subgroups were compared by Kaplan–Meier curves and log-rank tests. The independent prognostic values of the clinical characteristics of OS were evaluated by univariate Cox proportional hazard regression analyses. The prognostic abilities of the predictive models for OS were evaluated using receiver operating characteristic (ROC) curves (R package “timeROC”) and area under the curve (AUC) values. Differences between groups were compared by independent samples *t*-tests. *p* < 0.05 was considered statistically significant.

## Results

### Prognostic m6A-Related lncRNAs in LUAD Patients

Using the file downloaded from the TCGA website (https://portal.gdc.cancer.gov/), we identified 60,484 mRNAs including 23 m6A-related genes in the TCGA, and selected 14,087 lncRNAs from the mRNA dataset for follow-up analysis. We defined lncRNAs with expression levels that correlated with one or more of the 23 m6A-related genes as m6A-related lncRNAs by Pearson’s correlation analysis, according to the selection criteria R > |0.5|, *p* < 0.001. This identified 1,549 lncRNAs with expression levels significantly correlated with m6A-related genes. Using univariate Cox regression analysis of prognostic information and expression of m6A-related lncRNAs in TCGA datasets (*p* < 0.05), we identified 91 m6A-related lncRNAs with expression levels with significant impacts on OS among LUAD patients based on TCGA datasets. The workflow is shown in Figure 1A and the correlations between the 1,549 lncRNAs and 23 m6A-related genes according to the TCGA dataset are shown in Figure 1B. The results of univariate Cox analysis of the 91 prognostic m6A-related lncRNAs are shown in Supplementary Table S1 and Supplementary Figure S1A. We also compared the expression levels of the 91 lncRNAs between cancerous and normal tissues (Figure 1C and Supplementary Figure S1B).

### Consensus Clustering Identified Two Clusters of Patients With LUAD

**FIGURE 2:**
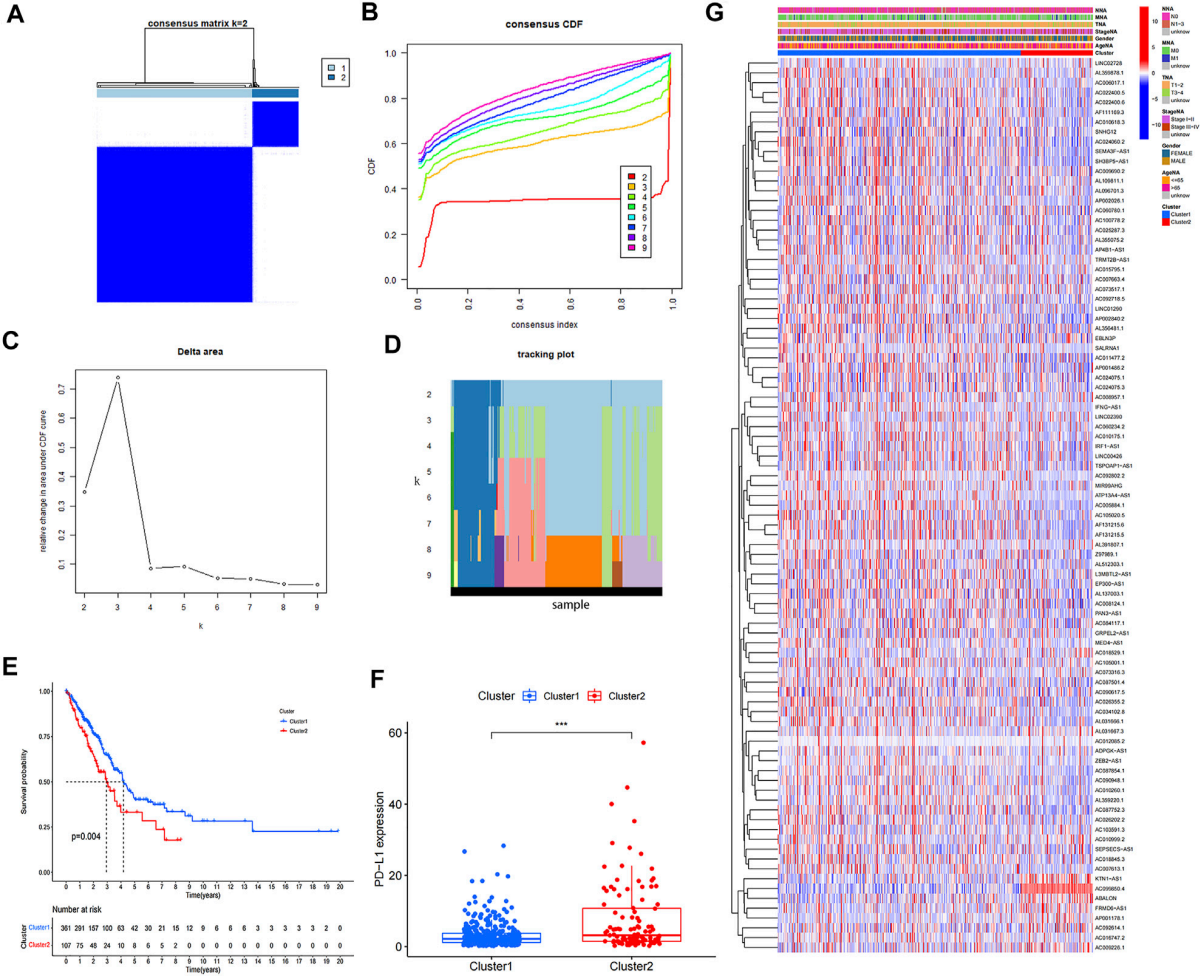
Consensus clustering of prognostic m6A-related lncRNAs. **(A)** Consensus clustering matrix for *k* = 2. **(B–D)** Consensus clustering cumulative distribution function (CDF) and relative change in area under CDF curve for *k* = 2–9. **(E)** Kaplan–Meier curves for two clusters in LUAD. **(F)** Expression of PD-L1 in two clusters (*p < 0.05; **p < 0.01; ***p < 0.001). **(G)** Heatmap of associations between expression levels of 91 prognostic m6A-related lncRNAs and clinicopathological features in the TCGA-LUAD dataset.

We confirmed the correlations between prognostic m6A-related lncRNAs by Pearson’s correlation analysis (Figure 2A). Highly correlated (|correlation coefficient| = 1, *p* < 0.05) prognostic m6A-related lncRNAs were: ADPGK-AS1, ZEB2-AS1, AC092614.1, AC015795.1, AC084117.1, AC090948.1, AC018529.1, AC010618.3, AL137003.1, AC010175.1, AF131215.5, KTN1-AS1, AC006017.1, L3MBTL2-AS1, AC026355.2, AC099850.4, LINC00426, AC009690.2, AC010999.2, GRPEL2-AS1, LINC02390, AC009226.1, AC025287.3, ABALON, AC090617.5, AC034102.8, FRMD6-AS1, AL359220.1, AC26202.2,AF111169.3, AC008957.1, EBLN3P, AC024075.3, AC073517.1, IFNG-AS1, IRF1-AS1, TSPOAP1-AS1, and AP4B1-AS1. We divided the TCGA-LUAD cohort based on expression of prognostic m6A-related lncRNAs into two subgroups using the “ConsensusClusterPlus” R package. The crossover in LUAD samples was lowest when we chose 2 as the consensus matrix k value (Figures 2B,C and D). Kaplan–Meier survival analysis identified survival differences between the two clusters (Figure 2E) and detected higher expression of PD-L1 in cluster2 compared with cluster1 (Figure 2F). The expression of 91 prognostic m6A-related lncRNAs in the two clusters are shown in a heatmap (Figure 2G). Expression levels of AF131215.6, ATP13A4-AS1, LINC01290, AC087854.1, AC011477.2, AC010618.3, AC010260.1, AC087752.3, SALRNA1, AP002026.1, AL137003.1, AC010175.1, AF131215.5, AC024075.1, AC007613.1, AC105020.5, LINC00426, SNHG12, AC034102.8, AC092718.5, AL359220.1, AP002840.2, AC022400.5, AF111169.3, MED4-AS1, AL512303.1, AC024060.2, AC024075.3, SEMA3F-AS1, AC005884.1, AC022400.6, AP001486.2, IFNG-AS1, PAN3-AS1, EP300-AS1, SH3BP5-AS1, TSPOAP1-AS1, and AP4B1-AS1 were all higher in cluster1 compared with cluster2, while KTN1-AS1, AC099850.4, AC009226.1, ABALON, and FRMD6-AS1 were lower in cluster 1 compared with cluster 2 (*p* < 0.01).

### Gene Set Enrichment Analysis Between the Two Clusters

**FIGURE 3:**
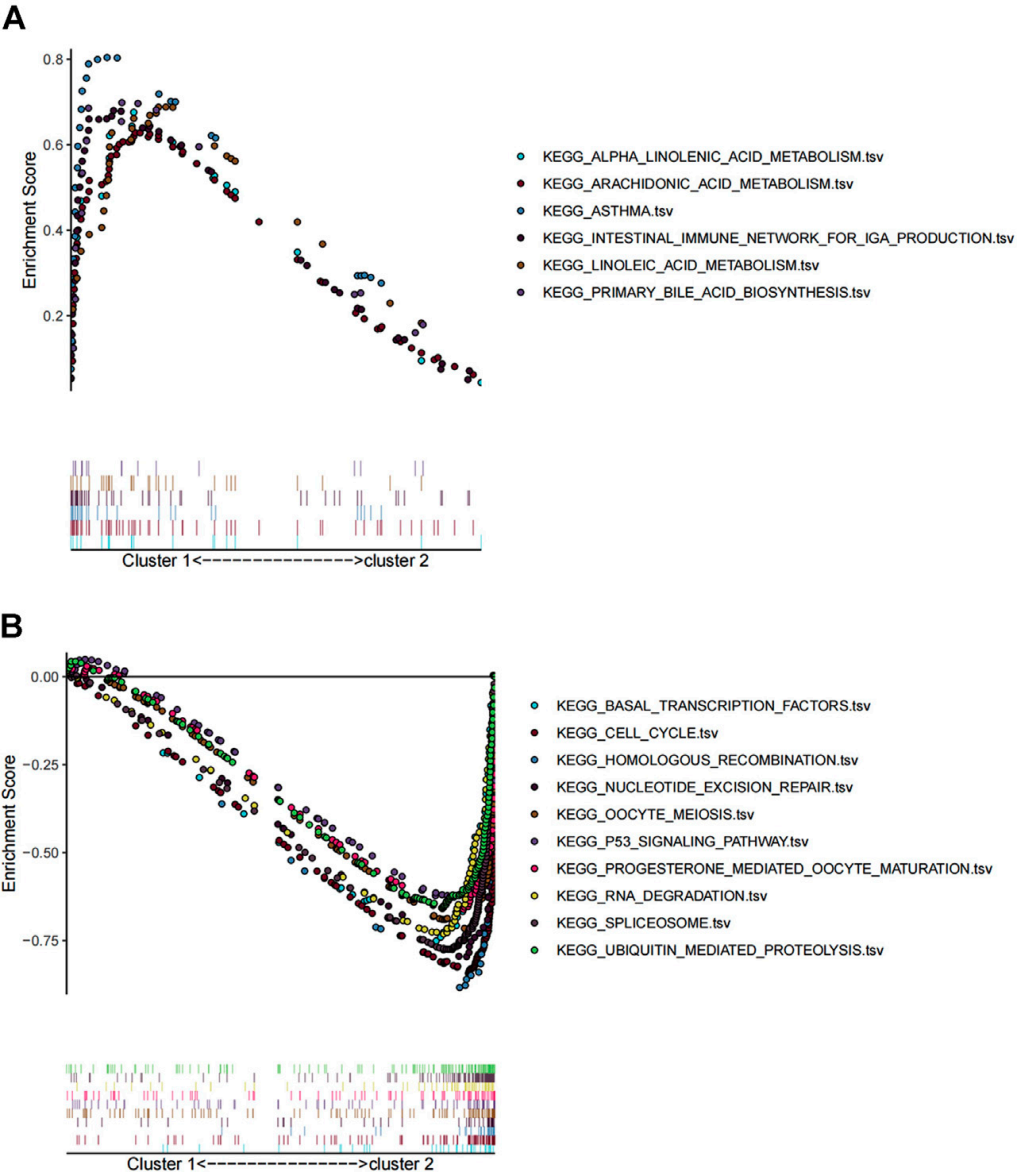
GSEA analysis of differentially expressed genes between cluster 1 and cluster 2. **(A)** KEGG analysis of upregulated pathway in cluster 1 and **(B)** downregulated pathway in cluster 2.

We performed GSEA based on “c2.cp.kegg.v6.2.symbols.gmt” gene sets combing differentially expressed genes between the two clusters, to identify differences in biological processes and pathways. GSEA showed that genes in cluster 1 were mainly enriched in alpha linolenic acid metabolism, arachidonic metabolism, asthma, intestinal immune network for IgA production, linoleic acid metabolism, and primary bile acid biosynthesis (Figure 3A). In contrast, genes in cluster 2 were primarily enriched in basal transcription factors, cell cycle, homologous recombination nucleotide excision repair, oocyte meiosis, P53 signaling pathway, progesterone-mediated oocyte maturation, RNA degradation, spliceosome, and ubiquitin-mediated proteolysis (Figure 3B).

### Immune Landscape and TME in LUAD Patients

**FIGURE 4:**
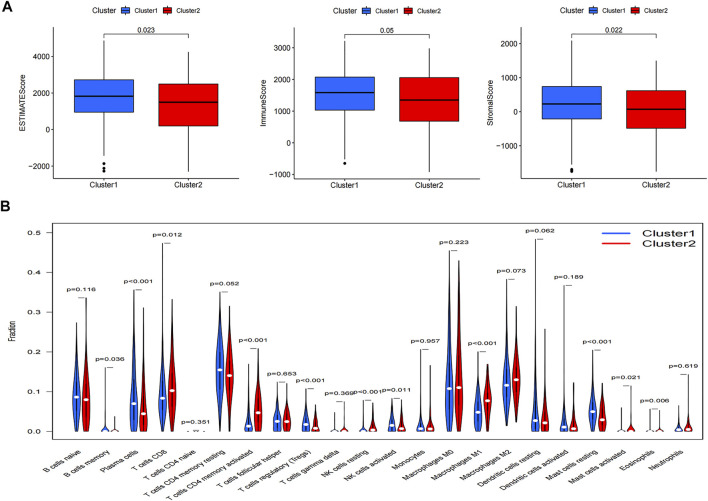
**(A)** Differences in ESTIMATE, immune, and stromal scores between the two clusters. **(B)** Differences in levels of infiltration between the two clusters.

We calculated the density and location of immune cells in LUAD samples using the ESTIMATE algorithm to provide immune, stromal, and ESTIMATE scores. The immune, stromal, and ESTIMATE scores were all higher in cluster 1 compared with cluster2 (Figure 4A), while TME was higher in cluster 2. We also analyzed the differences in 22 immune cell types using the CIBERSORT algorithm. Cluster 1 included more naïve B cells, plasma cells, regulatory T cells, activated natural killer (NK) cells, and resting mast cells than cluster 2. OS was also better in cluster 1, and cluster 2 had a worse prognosis (Figure 4B). Numbers of CD8 T cells, resting memory CD4 T cells, and M1 macrophages were also lower in cluster 1 compared with cluster 2 (Figure 4B). Box diagrams of the numbers of individual types of immune cells are shown in Supplementary Figures S2–S4.

### Prognostic Ability of 13 m6A-Related lncRNAs

**FIGURE 5:**
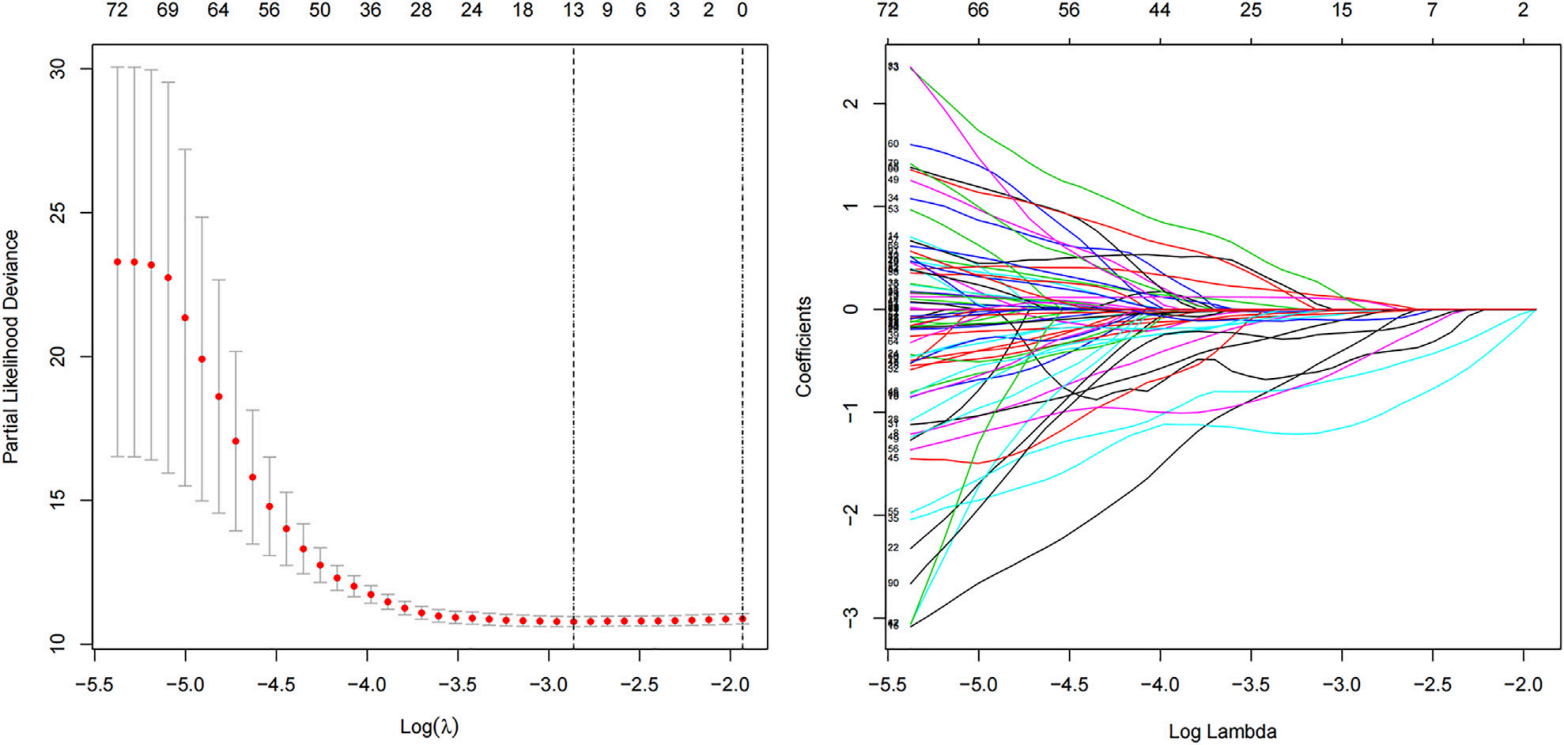
LASSO Cox regression analysis of prognostic m6A-related lncRNAs.

Ninety-one prognostic m6A-related lncRNAs were identified to establish a signature for prognosis prediction of LUAD using LASSO Cox regression. Characteristic of LUAD patients in train, test and all TCGA data set were shown in Table 1. We then used 13 m6A-related lncRNAs (ADPGK-AS1, AC103591.3, AC018529.1, AC010175.1, AC016747.2, AC007613.1, AC026355.2, ABALON, AC034102.8, AC073316.3, AL031667.3, AC005884.1 and TSPOAP1-AS1) to establish a risk model to calculate the risk score (Figure 5) as follows: Risk score = (−0.437) × expression quantity (Exp) of ADPGK-AS1 + (−0.004) × Exp of AC103591.3 + (−0.270) × Exp of AC018529.1 + (−0.017) × Exp of AC010175.1 + (−0.052) × Exp of AC016747.2 + (−1.080) × Exp of AC007613.1 + (−0.031) × Exp of AC026355.2 + 0.083 × Exp of ABALON + (−0.616) × Exp of AC034102.8 + (−0.456) × Exp of AC073316.3 + 0.075 × Exp of AL031667.3 + (−0.103) × Exp of AC005884.1 + (−0.212) × Exp of TSPOAP1-AS1.

**TABLE 1:** Characteristic of LUAD patients in train, test and all TCGA data set.

Characteristics		Train set (*n* = 236)	Test set (*n* = 232)	*p* value^a^	Total set (*n* = 468)
Age (IQR), year		67.0 (59.0, 7.3.0)	64.0 (59.0, 72.0)	0.229	66.0 (59.0, 72.0)
Gender	Male	116 (49.15%)	98 (42.24%)	0.133	214 (45.73%)
	Female	120 (50.85%)	134 (57.76%)		254 (54.27%)
Race	American Indian or Alaska native	0 (0%)	1 (0.43%)		1 (0.21%)
	Asian	6 (2.54%)	1 (0.43%)		7 (1.50%)
	Black or African American	23 (9.75%)	27 (11.64%)		50 (10.68%)
	White	182 (77.12%)	173 (74.57%)		355 (75.85%)
	Unknow	25 (10.59%)	30 (12.93%)		55 (11.75%)
Stage	Stage I	126 (53.39%)	127 (54.74%)	0.843	253 (54.06%)
	Stage II	57 (24.15%)	50 (21.55%)		107 (22.86%)
	Stage III	39 (16.53%)	36 (15.52%)		75 (16.03%)
	Stage IV	11 (4.66%)	14 (6.03%)		25 (5.34%)
	Unknown	3 (1.27%)	5 (2.16%)		8 (1.71%)
T	T1	80 (33.9%)	79 (34.05%)	0.426	159 (33.97%)
	T2	124 (52.54%)	124 (53.45%)		248 (52.99%)
	T3	23 (9.75%)	16 (6.9%)		39 (8.33%)
	T4	9 (3.81%)	10 (4.31%)		19 (4.06%)
	Unknown	0 (0%)	3 (1.29%)		3 (0.64%)
M	M0	158 (66.95%)	157 (67.67%)	0.848	315 (67.31%)
	M1	11 (4.66%)	13 (5.6%)		24 (5.13%)
	Unknown	67 (28.39%)	62 (26.72%)		129 (27.56%)
N	N0	153 (64.83%)	149 (64.22%)	0.395	302 (64.53%)
	N1	47 (19.92%)	39 (16.81%)		86 (18.38%)
	N2	32 (13.56%)	34 (14.66%)		66 (14.1%)
	N3	1 (0.42%)	1 (0.43%)		2 (0.43%)
	Unknown	3 (1.27%)	9 (3.88%)		12 (2.56%)
Smoking	Smoker	29 (12.29%)	37 (15.95%)	0.466	66 (14.1%)
	Non-smoker	200 (84.75%)	190 (81.9%)		390 (83.33%)
	Unknown	7 (2.97%)	5 (2.16%)		12 (2.56%)

Data are presented as the median (interquartile range) or No. (percentage).

Abbreviation: IQR, interquartile range.

a Comparison between groups (train and test set): Mann-Whitney test, or χ^2^ test or Fisher exact test.

**FIGURE 6:**
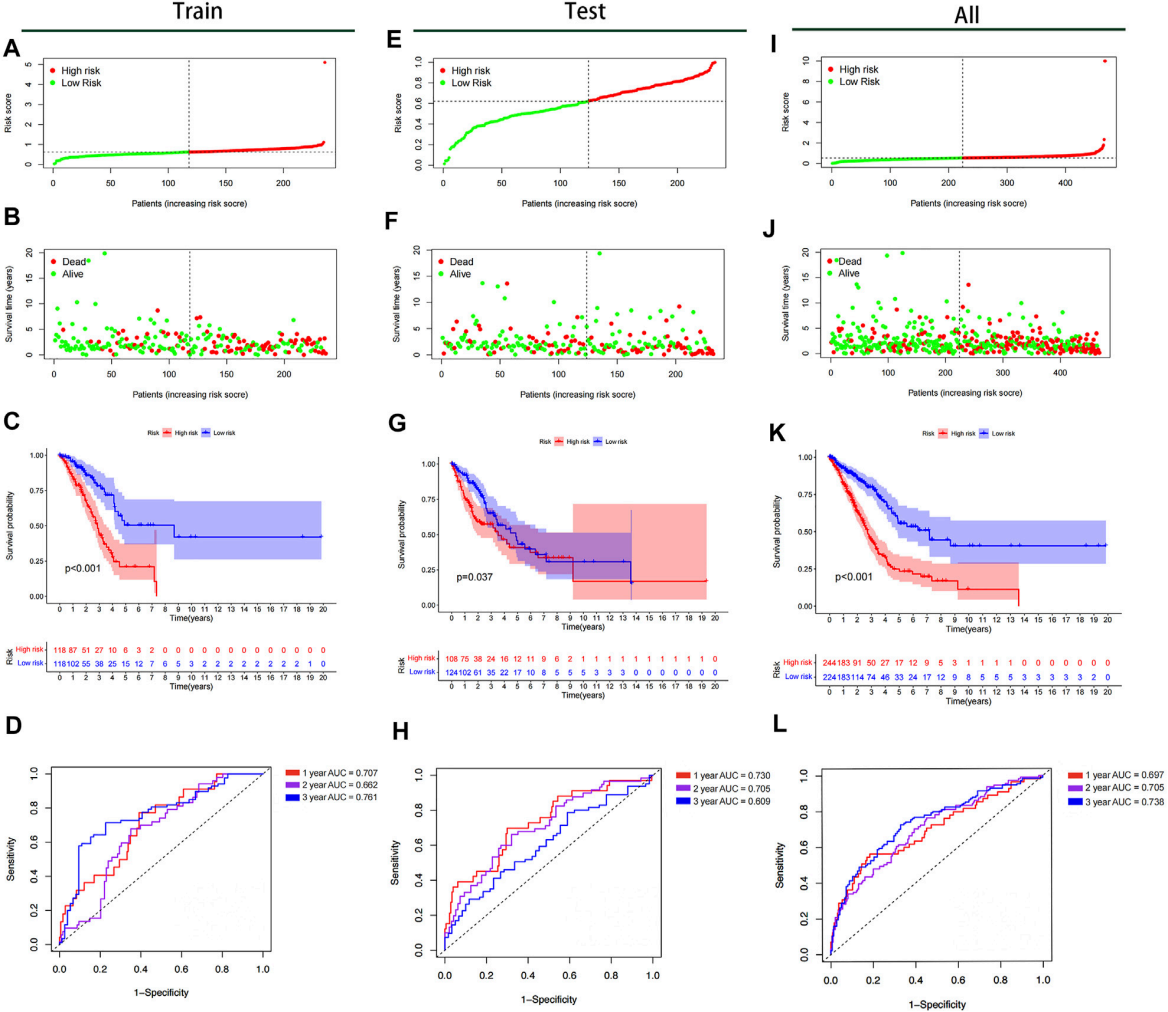
Kaplan–Meier survival analysis, risk score analysis, heatmap, and time–ROC curve analysis. **(A,B)** Risk score, **(C)** Kaplan–Meier curve, **(D)** time–ROC curve of prognostic m6A-related lncRNA signature in the training cohort. **(E,F)** Risk score, **(G)** Kaplan–Meier curve, **(H)** time–ROC curve of prognostic m6A-related lncRNA signature in the test cohort. **(I,J)** Risk score, **(K)** Kaplan–Meier curve, **(L)** time–ROC curve of prognostic m6A-related lncRNA signature in the all cohort.

**FIGURE 7:**
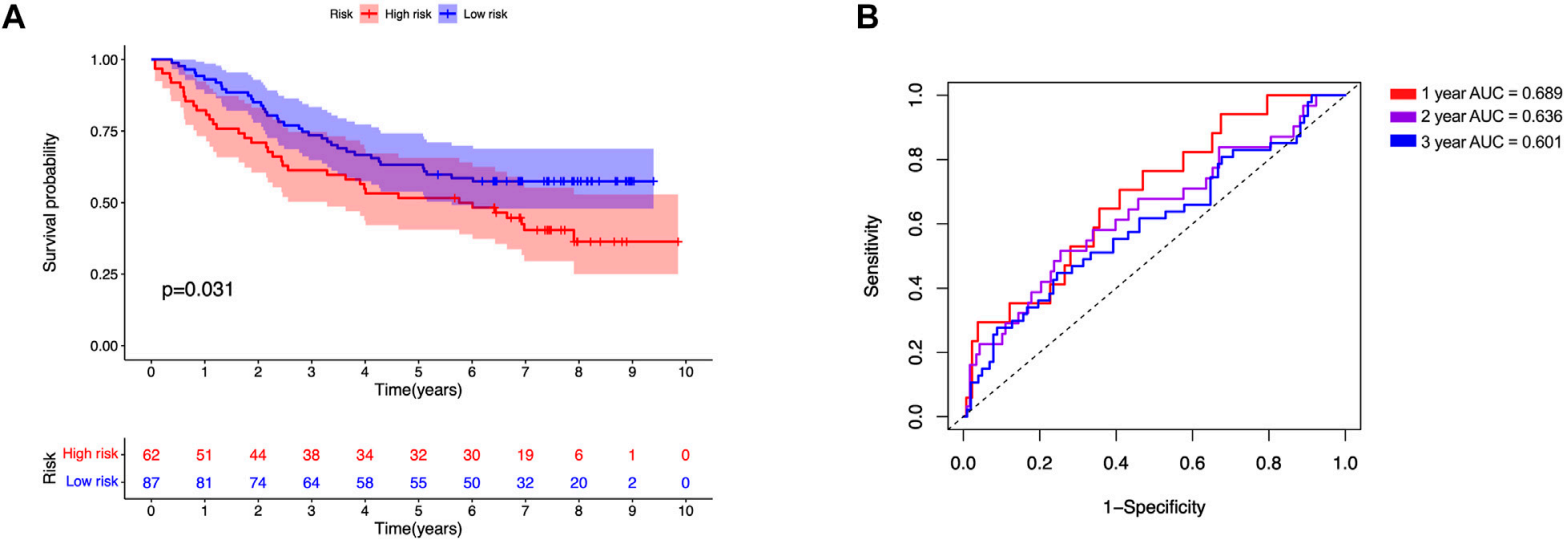
Validation of GSE11969 independent dataset. **(A)** Kaplan–Meier survival analysis, **(B)** time–ROC curve of prognostic m6A-related lncRNA signature in GSE11969 independent dataset.

All samples were subsequently classified as high-risk (*n* = 118) or low-risk (*n* = 118) according to the median risk scores. The risk score curve and survival status data for the two groups are shown in Figures 6A,B,E,F,I,J. The m6A-LPS was then verified in training, test and all datasets, and patients in the high-risk group had poorer OS than those in the low-risk group (*p* < 0.05; Figures 6C,G,K). The 3- and 6-years survival rates of LUAD patients in the low- and high-risk groups were 32.2 and 10.2%, and 22.9 and 2.5%, respectively. And the ROC curves demonstrated that m6A-LPS harbored a promising ability to predict OS in the train data set (1-year AUC = 0.707, 2-years AUC = 0.662, 3-years AUC = 0.761; Figure 6D) and test data set (1-year AUC = 0.730, 2-years AUC = 0.705, 3-years = 0.609; Figure 6H) and all data set (1-year AUC = 0.697, 2-years AUC = 0.705, 3-years = 0.738; Figure 6L). In addition, patients with LUAD in the GSE11969 data set were analyzed. Patients in the high-risk group had a lower survival rate than those in the low-risk group, and the 1, 2, and 3 years AUC were 0.689, 0.636 and 0.601, respectively (Figure 7).

Further, we compared lncRNAs based model for LUAD using the concordance index calculated by TCGA cohort (*n* = 468). The result shows that the concordance index of our model (0.667) was comparable to Song et al.’s (0.653), Zhang et al.’s (0.655), Ren et al.’s (0.645), Miao et al.’s (0.665) and Lu et al.’s (0.695) models (Song et al., 2020; Lu et al., 2021; Miao et al., 2021; Ren et al., 2021; Zhang et al., 2021), which is shown in Table 2.

**TABLE 2:** The concordance index of already existing lncRNAs based model for LUAD.

	This study	Song et al.	Zhang et al.	Miao et al.	Ren et al.	Lu et al.
Gene name	*ADPGK-AS1*	*AC018629.1*	*C20orf197*	*AC020915.2*	*CASC15*	*C5orf64*
	*AC103591.3*	*AC122134.1*	*LINC00319*	*AC245595.1*	*CRNDE*	*LINC01800*
	*AC018529.1*	*AC119424.1*	*AC090286.1*	*FAM83A-AS1*	*LINC01137*	*LINC00968*
	*AC010175.1*	*AL138789.1*	*AC004485.1*	*AL606834.1*	*CYP1B1-AS1*	*LINC01352*
	*AC016747.2*		*AL355916.1*	*LINC00941*		*PGM5-AS1*
	*AC007613.1*		*LINC00941*	*AC026369.3*		*LINC02097*
	*AC026355.2*		*AC119150.1*			*DEPDC1-AS1*
	*ABALON*		*AC025419.1*			*WWC2-AS2*
	*AC034102.8*		*AC034223.2*			*SATB2-AS1*
	*AC073316.3*		*AC073651.1*			*LINC00628*
	*AL031667.3*		*AC007406.4*			*LINC01537*
	*AC005884.1*		*LINC02320*			*LM O 7DN*
	*TSPOAP1-AS1*		*AC097504.2*			
			*AL161431.1*			
Concordance index	0.667	0.653	0.655	0.665	0.645	0.695

The expression differences of the m6A-related lncRNAs in m6A-LPS are displayed in Figures 7A,C. The heatmap showed that ABALON was upregulated in high-risk compared with low-risk patients, whereas ADPGK-AS1, AC103591.3, AC018529.1, AC010175.1, AC016747.2, AC007613.1, AC026355.2, AC034102.8, AC073316.3, AC005884.1, and TSPOAP1-AS1 were downregulated in the high-risk compared with the low-risk group (*p* < 0.001).

### Independent Prognostic Factors

**FIGURE 8:**
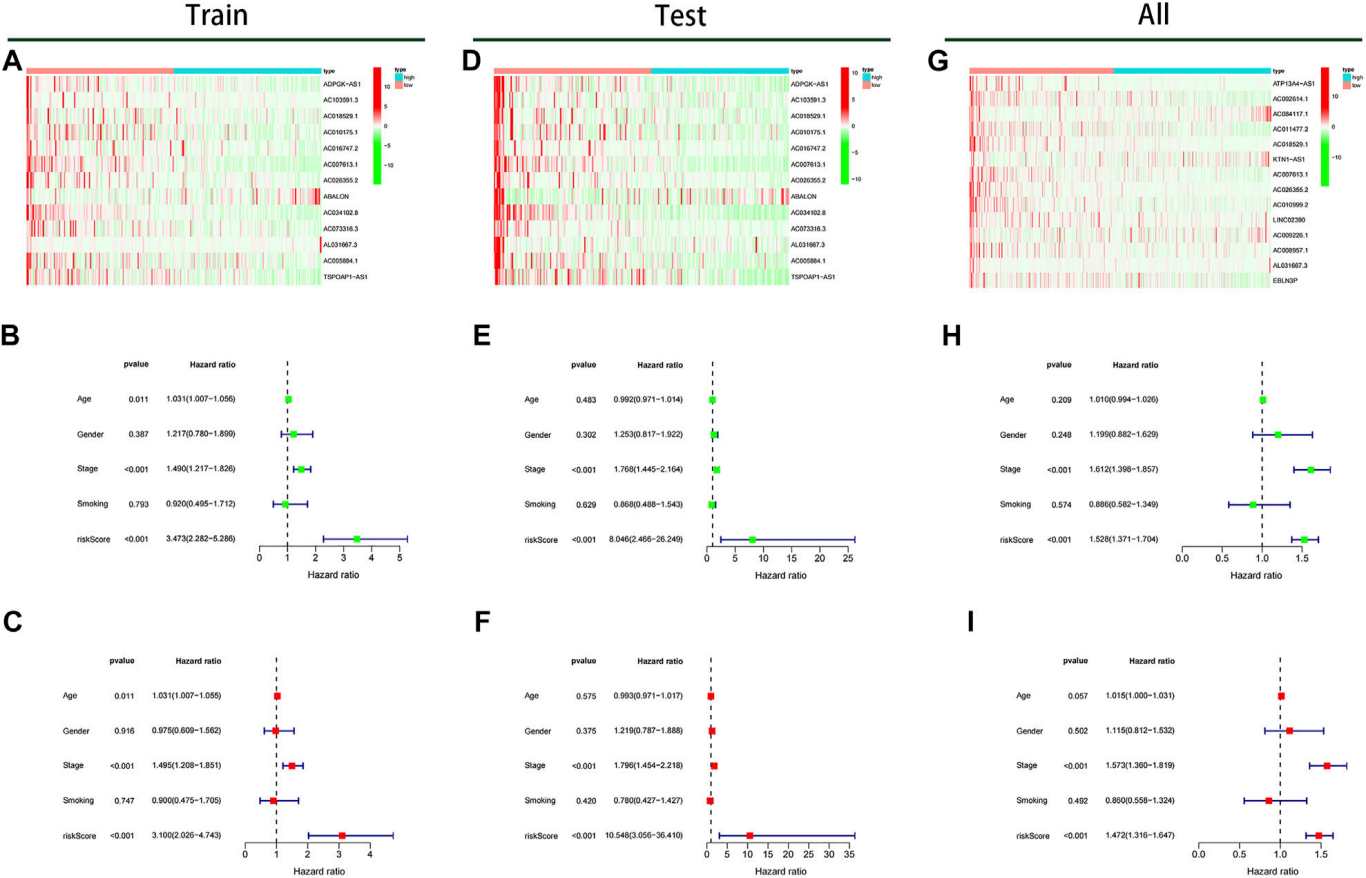
The expression of 13 prognostic m6A-lncRNAs and analysis of prognostic factors. **(A)** Heatmap of expression of prognostic m6A-lncRNAs in the training cohort. Univariate **(B)** and multivariate **(C)** prognostic factors analysis forest plot of the model in the training cohort. **(D)** Heatmap of expression of prognostic m6A-lncRNAs in the test cohort. Univariate **(E)** and multivariate **(F)** prognostic factors analysis forest plot of the model in the test cohort. **(G)** Heatmap of expression of prognostic m6A-lncRNAs in the TCGA cohort. Univariate **(H)** and multivariate **(I)** prognostic factors analysis forest plot of the model in the TCGA cohort.

The expressions of 13 prognostic m6A-related lncRNAs between high- and lowrisk subgroups were shown in Figures 8A,D,G. Cox regression analysis identified risk score and stage as independent prognostic predictive factors for LUAD patients in the training dataset (*p* < 0.001) [hazard ratio (HR) of risk score 3.473 (2.282–5.286) and HR of stage 1.490 (1.217–1.826); Figure 8B]. Similarly, the risk score and stage were also identified as independent prognostic predictive factors for LUAD patients using Cox regression analysis based on the test dataset [HR of risk score 8.046 (2.466–26.249) and HR of stage 1.768 (1.445–2.164); Figure 8E] and all data set [HR of risk score 1.528 (1.371–1.740) and HR of stage 1.612 (1.398–1,857); Figure 8H]. Besides, after adjustment for age, smoking, gender and stage, risk score calculated by our model was also identified as independent prognostic predictive factors for LUAD patients, with consistent results in training, test, and all data set (Figures 8C,F,I).

### Stratification of m6A-LPS

**FIGURE 9:**
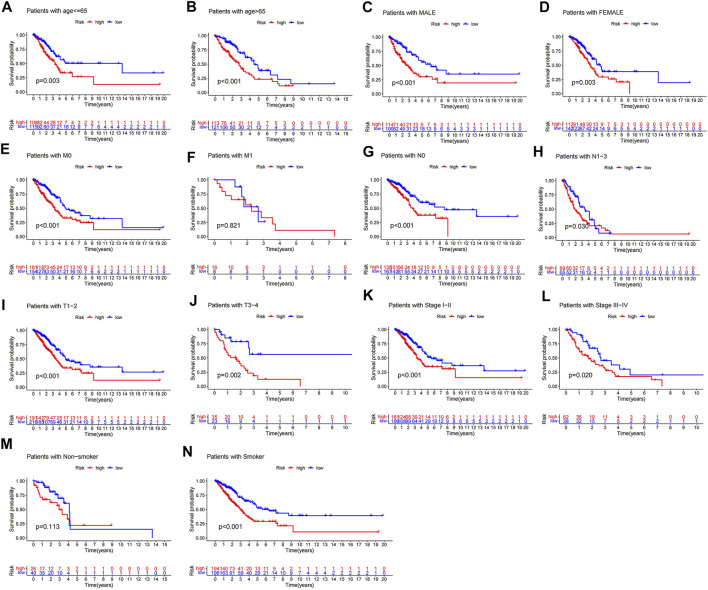
Stratification analysis of survival according to clinicopathological characteristics. **(A,B)** Survival analysis of all patients adjusted to age, **(C,D)** sex, **(E,F)** M stage, **(G,H)** N stage, **(I,J)** T stage, **(K,L)** pathological stage, and **(M,N)** somoking between high and low-risk groups.

The above results suggested that m6A-LPS could predict OS in various subgroups of patients with LUAD, by stratification analysis. Low-risk patients had better OS than high-risk patients in all age groups (Figures 9A,B). Likewise, sex did not affect the ability of m6A-LPS to predict OS in LUAD patients (Figures 9C,D). Additionally, m6A-LPS could predict OS in LUAD patients with stage M0, all N stages, all T stages, and all stage subgroups apart from stage M1 (limited by insufficient sample size) (Figures 9E–L). The results show that m6A-LPS could predict OS in smoker subgroups of patients with LUAD. But the patients of survival time for more than 4 years in the non-smoker subgroup (*n* = 66) were only 7, which contribute to the survival rates estimated inaccurately and the stern of survival curve overlapped (Figure 9).

**FIGURE 10:**
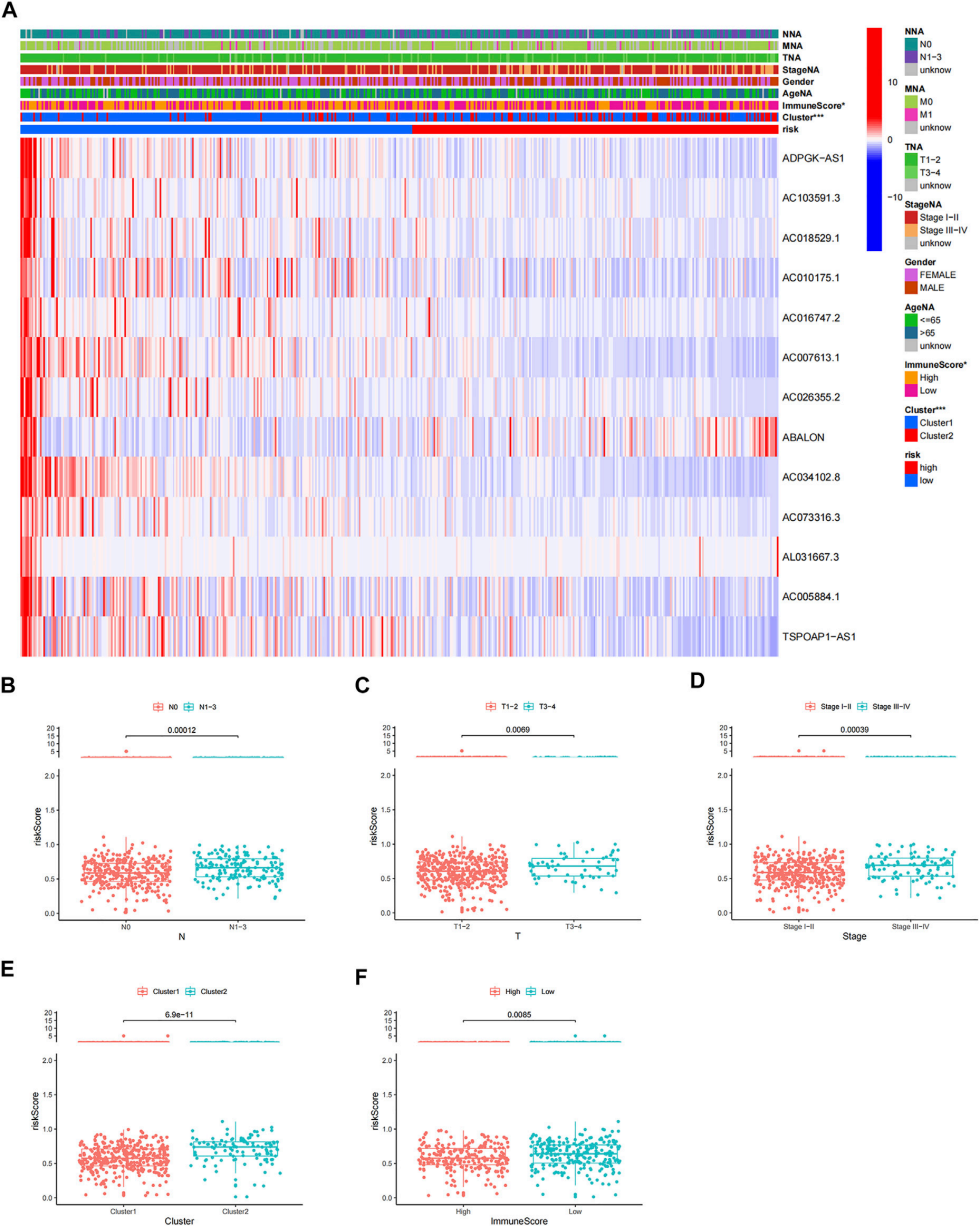
Correlation analysis of risk score and clinicopathological characteristics. **(A)** Heatmap of expression of prognostic m6A-related lncRNAs in relation to age, sex, TMN stage, lncRNA-clusters, pathological stage, and immune score. Variance analysis of risk scores according to **(B)** N stage, **(C)** T stage, **(D)** pathological stage, **(E)** two clusters, and **(F)** different levels of immune score.

Patients in the high-risk group had lower immune scores than those in the low-risk group, and patients in cluster 2 had higher risk scores than those in cluster 1 (Figures 10A,E,F). Patients with LUAD stage N1–3, T3–4, and stage III–IV had higher risk scores than those with N0, T1–2, and stage I–II respectively (Figures 10B–D), but there were no differences in risk scores in relation to age, sex, and M stage (Supplementary Figures S5A–C). These results suggest that the risk score reflected the malignant stage and TME.

### Levels of Immune Cells Based on Risk Score

**FIGURE 11:**
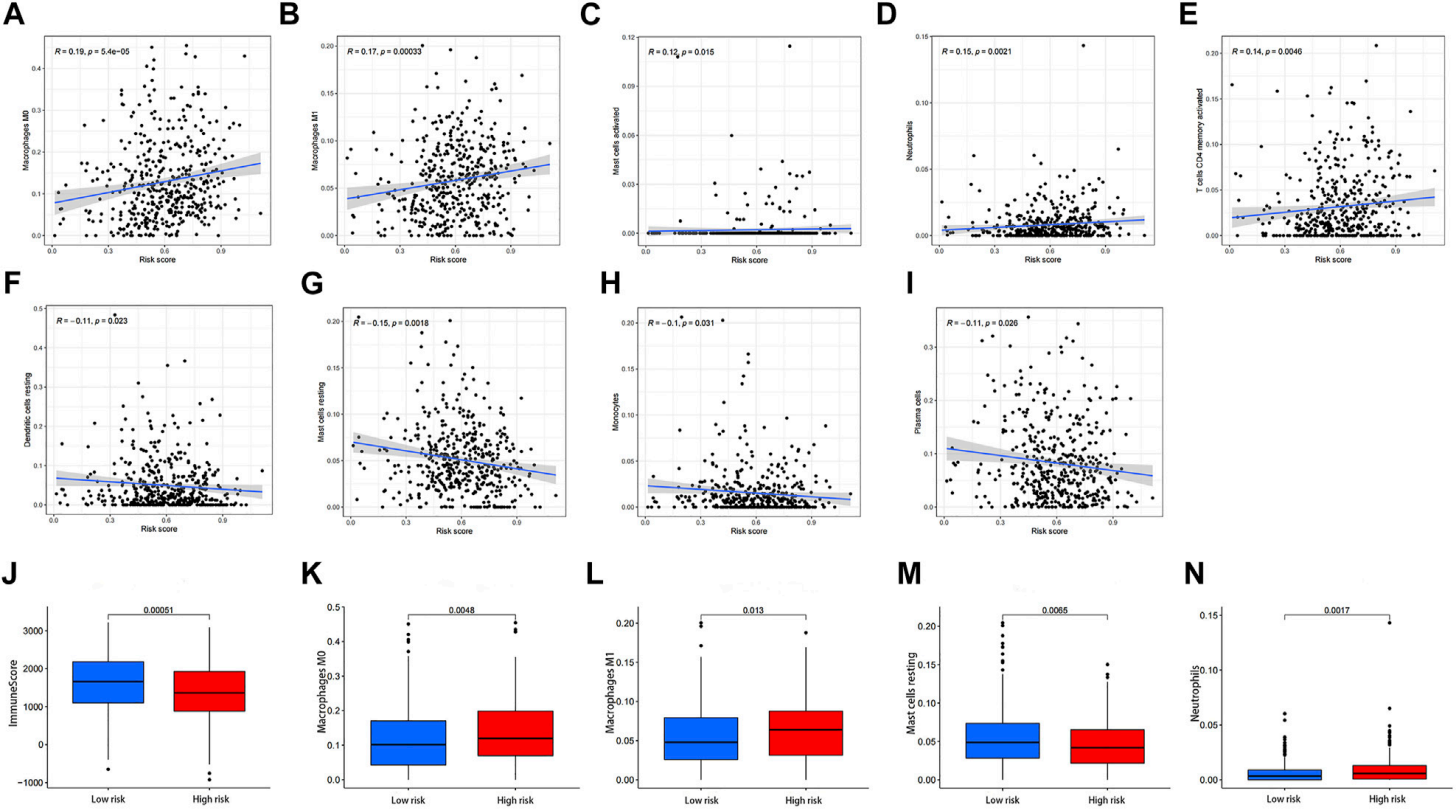
Correlation analysis of risk score and immune cells. **(A)** M0 macrophages, **(B)** M1 macrophages, **(C)** activated mast cells, **(D)** neutrophils, **(E)** activated memory CD4 T cells, **(F)** resting dendritic cells, **(G)** resting mast cells, **(H)** monocytes, and **(I)** plasma cells. **(J)** The difference of immuneScore, **(K)** M0 macrophages, **(L)** M1 macrophages, **(M)** resting mast cells **(N)** neutrophils between high- and low-risk subgroups.

We found a significant positive correlation between risk score and numbers of M0 and M1 macrophages, activated mast cells, neutrophils, and activated CD4 memory T cells (Figures 11A–E). There were also significant negative correlations between risk score and numbers of resting dendritic cells, resting mast cells, monocytes, and plasma cells (Figures 11F–I). These results suggested that the number of immune cells might influence tumor malignancy and prognosis. The difference of immuneScore M0 macrophages M1 macrophages, resting mast cells and neutrophils between high- and low-risk subgroups were shown in (Figures 11J–N).

### Analysis of Online Databases

**FIGURE 12:**
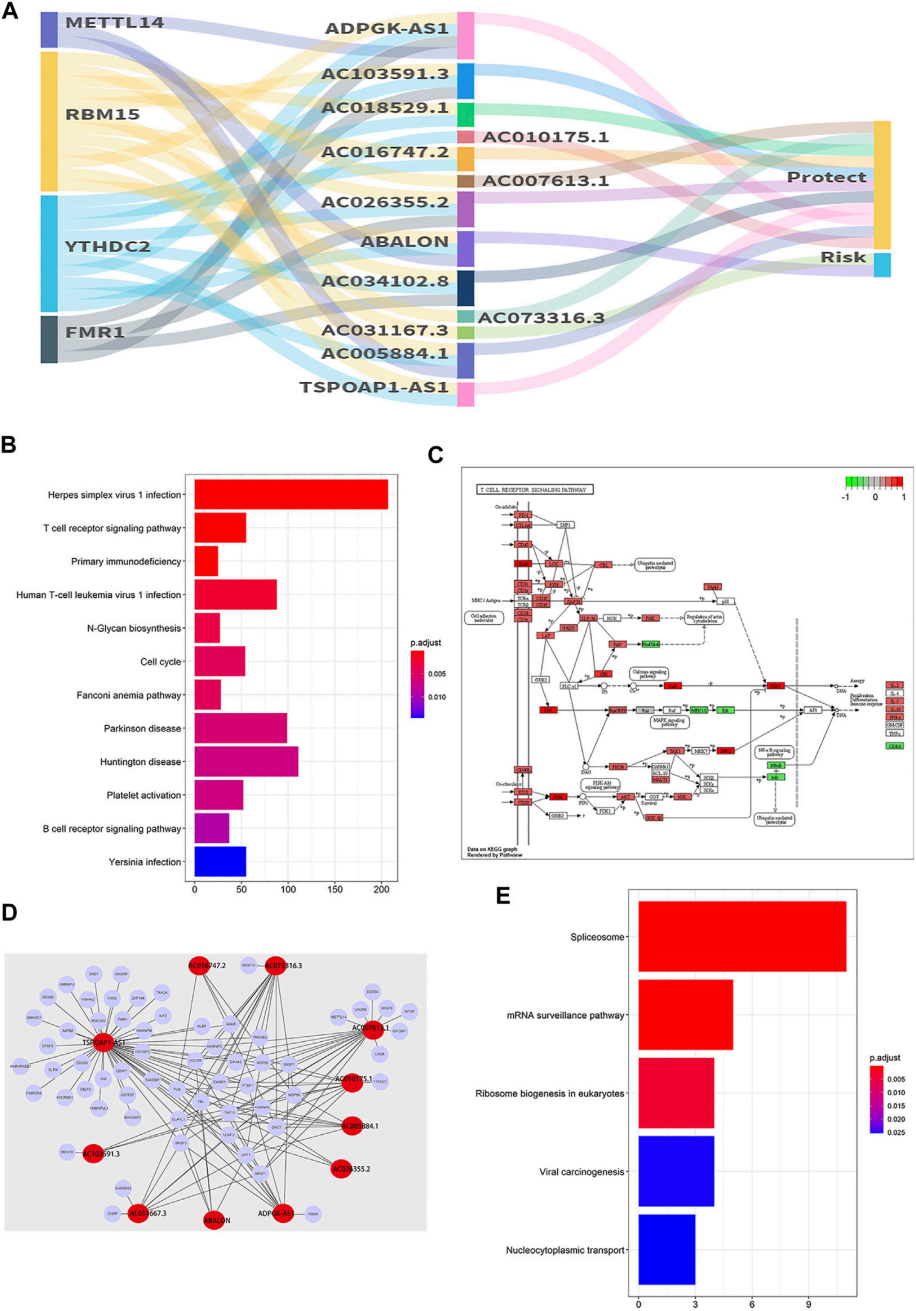
The co-expression network and gene enrichment analysis. **(A)** The constructed m6A-related prognostic lncRNAs and m6A genes co-expression network was visualized using the Sankey diagram. **(B)** KEGG analysis of target genes (based on co-expression) of 13 m6A-related lncRNAs. **(C)** The pathway of the T cell receptor signaling pathway (hsa04660). **(D)** The network is based on 13 prognostic m6A-related lncRNAs and the potential target genes identified by the RNA binding protein (RBP)-lncRNA module in Starbase. **(E)** KEGG analysis of 134 potential target genes (based on Starbase database) of 13 m6A-related lncRNAs.

The relationships of m6A RNA methylation regulators, 13 m6A-related prognostic lncRNAs and risk are shown in Figure 12A. And the results of StarBase and GEPIA verifid the correlation between 13 prognostic lncRNAs and m6A RNA methylation regulators (Supplementary Figures S6, S7).

### Gene Enrichment Analysis of the Targets of 13 m6A-Related Prognostic lncRNAs

The gene enrichment analysis for targets of 13 prognostic lncRNAs was conducted and the results were shown in Figure 12B. Interestingly, the PI3K/AKT of T cell receptor signaling pathway with high enrichment score was positively correlated with ABALON and AL031667.3 of high-risk prognostic lncRNAs (*p* < 0.001) (Figure 12C). In addition, 134 potential target genes were identified based on the interaction of RBP and lncRNA in the Starbase database (Figure 12D), and the result of gene enrichment analysis showed that genes were primarily enriched in the spliceosome, mRNA surveillance, ribosome biogenesis in eukaryotes, viral carcinogenesis and nucleocytoplasmic transport pathway (Figure 12E).

### The Potential m6A Modification Sites of 13 m6A-Related lncRNAs

The predictions of potential m6A modification sites on 13 m6A-related lncRNAs were conducted and the results were shown in Supplementary Figure S8. The Supplementary Table S2 indicated the detailed information of these potential m6A modification sites.

## Discussion

LUAD is globally associated with high morbidity and a poor prognosis, presenting major challenges in terms of screening, diagnosis, and therapy (Denisenko et al., 2018). The carcinogenesis and development of LUAD involve major and varied molecular abnormalities that may provide potential diagnostic and therapeutic targets for LUAD. Numerous recent studies have highlighted the crucial role of aberrant m6A RNA methylation in cancer (Chen and Wong, 2020; Zhao et al., 2020). Furthermore, accumulating evidence has indicated that m6A modification and lncRNAs are involved in regulating the carcinogenesis, progression, and prognosis of tumors through lncRNAs endogenously targeting m6A regulators (Tu et al., 2020). Therefore, we established an m6A-related lncRNA signature to explore potential prognostic markers and therapeutic targets in LUAD patients.

We enrolled 486 patients with LUAD from TCGA datasets and identified 91 m6A-related lncRNAs as prognostic signatures for OS in LUAD patients. The TCGA-LUAD cohort was divided into two clusters by consensus expression of prognostic m6A-related lncRNAs. We showed that OS was poorer in patients in cluster 2 compared with cluster 1, indicating that the landscape of prognostic m6A-related lncRNAs affected the prognosis of LUAD patients. PD-L1 expression was also higher in cluster 2 than in cluster 1. Previous studies revealed that multiple lncRNAs indirectly regulated PD-L1 expression to impact the survival of cancer patients (Tian et al., 2021a; Tian et al., 2021b; Mu et al., 2021). We speculated that the expression profile of prognostic m6A-related lncRNAs in cluster 2 might increase PD-L1 expression, resulting in worse OS. Previous studies also reported that the effect of first-line treatment with the PD-1 inhibitor pembrolizumab was superior to platinum-doublet chemotherapy in patients with non-small-cell lung cancer and high PD-L1 expression (Aguilar et al., 2019), suggesting that the PD-1 inhibitor pembrolizumab might be an effective therapy for LUAD patients in cluster 2. Furthermore, LUAD patients in cluster 1 had higher immune, stromal, and ESTIMATE scores compared with cluster 2, indicating that the TME in cluster 1 was superior to cluster 2. GSEA revealed that the differentially expressed genes in cluster 2 were enriched in basal transcription factors, cell cycle, homologous recombination nucleotide excision repair, oocyte meiosis, P53 signaling pathway, progesterone-mediated oocyte maturation, RNA degradation, spliceosome, and ubiquitin-mediated proteolysis pathways. Among these, tumors have been shown to use homologous recombination nucleotide excision repair to protect DNA from serious damage, rendering the single-agent treatment ineffective (Zhu et al., 2021), while the ubiquitin-mediated proteolysis pathway has been shown to be involved in drug resistance in NSCLC (Sun et al., 2019).

Several studies have indicated that m6A regulators promote the malignant progression of multiple tumors by modifying specific lncRNAs (Lan et al., 2019; Ni et al., 2019; Yang et al., 2020). Further, we used specific lncRNAs to predict the risk of LUAD prognosis and progression, and enrolled 13 of the 91 m6A-related prognostic lncRNAs into the m6A-LPS by LASSO Cox regression, to improve the prediction of OS among LUAD patients. We classified LUAD patients into low- and high-risk subgroups according to the median, and showed that high-risk was correlated with poorer OS and TME. The m6A-LPS risk score was identified as an independent risk factor for OS using multivariate Cox regression analysis. Importantly, the risk score for cluster 2 with poor OS was higher than that for cluster 1 with longer OS, thus confirming the consistency of two methods for predicting prognosis in patients with LUAD. This prognostic signature was also able to predict the survival outcomes of LUAD patients by ROC curve analysis (1-year AUC = 0.707, 2-years AUC = 0.662, 3-years AUC = 0.761 and 1-year AUC = 0.730, 2-years AUC = 0.705, 3-years AUC = 0.609 in training and test datasets, respectively, *p* < 0.05). The m6A-LPS included ADPGK-AS1, AC103591.3, AC018529.1, AC010175.1, AC016747.2, AC007613.1, AC026355.2, ABALON, AC034102.8, AC073316.3, AL031667.3, AC005884.1, and TSPOAP1-AS1. High expression of TSPOAP1-AS1 has been associated with a better prognosis in patients with pancreatic ductal adenocarcinoma (Giulietti et al., 2018), consistent with the results in LUAD, while TSPOAP1-AS1 showed significantly higher methylation levels in pancreatic ductal adenocarcinoma compared with normal samples. In addition, TSPOAP1-AS1 lncRNA negatively modulated type I interferon signaling to facilitate influenza A virus replication (Wang et al., 2019). However, studies investigating the interactions between lncRNAs and m6A-related genes in LUAD are lacking. The current results thus identified potential prognostic lncRNAs targeted by m6A regulators, and provided clues to their potential function in the development and progression of LUAD. The results indicated that the target gene of 13 m6A-related lncRNAs mainly enriched in T cell receptor signaling pathway, N-Glycan biosynthesis, Cell cycle, Fanconi anemia pathway and Platelet activation, which were associated with metastasis and progression of cancer according to current studies (Lange et al., 2014; Nalepa and Clapp, 2018; Mammadova-Bach et al., 2020). Interestingly, the PI3K/AKT signaling of T cell receptor signaling pathway with high enrichment score was positively correlated with ABALON and AL031667.3 of high-risk prognostic lncRNAs, and many evidence indicated PI3K/AKT signaling activated promoted EMT, tumorigenesis, progression and metastasis of LUAD (Wei et al., 2019; Zhu et al., 2019; Xue et al., 2020; Liu et al., 2021). Thus, we speculated that ABALON and AL031667.3 may promote LUAD progression and metastasis by activating PI3K/AKT signaling and led to a poor prognosis.

Increasing studies have examined the TME and levels of tumor-infiltrating lymphocytes in relation to the prognosis in patients with NSCLC (Zhuang et al., 2010; Schalper et al., 2015). The results of the current study showed that the ESTIMATE, immune, and stromal scores were significantly correlated with the expression of m6A-related-lncRNA patterns. Specifically, the infiltration of some immune cells (memory B cells, plasma cells, regulatory T cells, resting mast cells, and activated NK cells) was significantly increased while the infiltration of other immune cells (CD8 T cells, activated CD4 memory T cells, M1 macrophages, activated mast cells, eosinophils, and resting NK cells) was significantly decreased in patients in cluster 1 compared with cluster 2. Similarly, the risk score was positively correlated with M0 and M1 macrophages, activated mast cells, neutrophils, and activated CD4 memory T cells, and negatively correlated with resting dendritic cells, resting mast cells, monocytes, and plasma cells. These results suggest that higher proportions of M1 macrophages, activated mast cells, and activated memory CD4 T cells, and lower proportions of resting mast cells, monocytes, and plasma cells might increase the risk of a poor prognosis in patients with LUAD. Higher immunosuppression and lower immunoreactivity in TME may result in a poorer prognosis among high-risk LUAD patients (Xu et al., 2020). Investigating the characteristics of TME cell infiltration in LUAD patients in relation to different m6A-related-lncRNAs patterns may thus facilitate the development of individualized novel immunotherapies.

However, our study has some limitations. First, this model was only validated in TCGA and GSE11969, and more external validation based on RNA-seq cohorts is needed in the future to evaluate whether it can be applied to clinical patients. In addition, we only preliminarily explored the signaling pathways involved in the targets of 13 m6A-related prognostic lncRNAs and the correlation of m6A-related lncRNAs with immunity, however, the specific mechanism of m6A-related lncRNAs in LUAD and their interconnection with immunity and m6A regulators are not yet fully understood, and more experimental studies are needed to validate our findings.

In summary, the current analyses revealed that different m6A-related-lncRNA patterns play an important role in the prognosis and in the heterogeneity and complexity of TME in patients with LUAD. We constructed a 13 m6A-related-lncRNAs signature prognostic model that could predict the clinical progression and prognostic risk of LUAD. These may thus be valuable candidate diagnostic and prognostic biomarkers and potential therapeutic targets for LUAD. Although further studies based on independent lung cancer cohorts are needed to ascertain the functions of lncRNAs and their interactions with m6A-related genes, the current results suggest that m6A-related-lncRNAs represent a novel avenue of research for tumor biomarkers.

## Data Availability

Publicly available datasets were analyzed in this study. This data can be found here: http://cancergenome.nih.gov/.
