# Supplementary material for: Relationships of N6-Methyladenosine-Related Long Non-Coding RNAs With Tumor Immune Microenvironment and Clinical Prognosis in Lung Adenocarcinoma
Source: Front Genet. 2021 Oct 20;12:714697. doi: 10.3389/fgene.2021.714697 (PMC8585518; doi:10.3389/fgene.2021.714697)
Supplement: Supplementary file 2 [file Table1.DOCX]

**Table S1** Detailed information for m6A-related lncRNAs significantly associated with OS in LUAD

| lnc-RNA | HR | HR.95L | HR.95H | P-value |
| --- | --- | --- | --- | --- |
| ADPGK-AS1 | 0.03 | 0.00 | 0.37 | 0.01 |
| AF131215.6 | 0.68 | 0.50 | 0.92 | 0.01 |
| ZEB2-AS1 | 0.14 | 0.03 | 0.82 | 0.03 |
| ATP13A4-AS1 | 0.88 | 0.79 | 0.99 | 0.03 |
| AC103591.3 | 0.87 | 0.77 | 1.00 | 0.05 |
| LINC01290 | 0.63 | 0.40 | 1.00 | 0.05 |
| AC018845.3 | 0.43 | 0.22 | 0.86 | 0.02 |
| AC092614.1 | 0.56 | 0.32 | 1.00 | 0.05 |
| AL109811.1 | 0.17 | 0.03 | 0.94 | 0.04 |
| AC015795.1 | 0.18 | 0.04 | 0.86 | 0.03 |
| AC084117.1 | 1.10 | 1.00 | 1.22 | 0.05 |
| AC087854.1 | 0.36 | 0.14 | 0.92 | 0.03 |
| AC011477.2 | 0.85 | 0.74 | 0.97 | 0.01 |
| AL391807.1 | 0.13 | 0.02 | 0.64 | 0.01 |
| AC090948.1 | 0.74 | 0.56 | 0.97 | 0.03 |
| AC018529.1 | 0.43 | 0.19 | 0.96 | 0.04 |
| AC087501.4 | 0.46 | 0.25 | 0.85 | 0.01 |
| AC010618.3 | 0.57 | 0.33 | 0.99 | 0.05 |
| AC010260.1 | 0.45 | 0.21 | 0.93 | 0.03 |
| AC087752.3 | 0.69 | 0.53 | 0.90 | 0.01 |
| AC060780.1 | 0.84 | 0.71 | 0.99 | 0.04 |
| AL359878.1 | 0.31 | 0.12 | 0.79 | 0.01 |
| SALRNA1 | 0.51 | 0.26 | 0.98 | 0.04 |
| AP002026.1 | 0.50 | 0.29 | 0.85 | 0.01 |
| AC060234.2 | 0.14 | 0.02 | 0.82 | 0.03 |
| AL137003.1 | 0.79 | 0.65 | 0.96 | 0.02 |
| TRMT2B-AS1 | 0.34 | 0.12 | 0.93 | 0.04 |
| AC010175.1 | 0.58 | 0.34 | 0.98 | 0.04 |
| AF131215.5 | 0.73 | 0.57 | 0.93 | 0.01 |
| AC024075.1 | 0.81 | 0.69 | 0.94 | 0.01 |
| AC016747.2 | 0.61 | 0.38 | 0.99 | 0.05 |
| KTN1-AS1 | 1.43 | 1.05 | 1.93 | 0.02 |
| AL355075.2 | 0.82 | 0.68 | 0.99 | 0.04 |
| AC105001.1 | 0.24 | 0.06 | 0.97 | 0.05 |
| AC007613.1 | 0.18 | 0.05 | 0.66 | 0.01 |
| AC007663.4 | 0.58 | 0.35 | 0.95 | 0.03 |
| AC006017.1 | 0.69 | 0.48 | 0.98 | 0.04 |
| L3MBTL2-AS1 | 0.39 | 0.20 | 0.77 | 0.01 |
| AC026355.2 | 0.88 | 0.80 | 0.97 | 0.01 |
| AC105020.5 | 0.45 | 0.22 | 0.91 | 0.03 |
| AC099850.4 | 1.03 | 1.01 | 1.06 | 0.00 |
| LINC02728 | 0.20 | 0.05 | 0.76 | 0.02 |
| LINC00426 | 0.62 | 0.42 | 0.93 | 0.02 |
| AC009690.2 | 0.48 | 0.25 | 0.95 | 0.03 |
| AC010999.2 | 0.17 | 0.05 | 0.57 | 0.00 |
| GRPEL2-AS1 | 0.37 | 0.14 | 0.97 | 0.04 |
| AC092802.2 | 0.27 | 0.08 | 0.93 | 0.04 |
| LINC02390 | 0.24 | 0.08 | 0.76 | 0.01 |
| AC009226.1 | 1.77 | 1.02 | 3.06 | 0.04 |
| AC025287.3 | 0.72 | 0.52 | 1.00 | 0.05 |
| SNHG12 | 0.94 | 0.90 | 0.99 | 0.01 |
| ABALON | 1.39 | 1.05 | 1.83 | 0.02 |
| Z97989.1 | 0.50 | 0.25 | 0.99 | 0.05 |
| AC090617.5 | 0.76 | 0.62 | 0.93 | 0.01 |
| AC034102.8 | 0.38 | 0.20 | 0.71 | 0.00 |
| AC073316.3 | 0.28 | 0.11 | 0.75 | 0.01 |
| FRMD6-AS1 | 2.41 | 1.23 | 4.72 | 0.01 |
| AL096701.3 | 0.42 | 0.19 | 0.90 | 0.03 |
| AC092718.5 | 0.56 | 0.32 | 0.98 | 0.04 |
| AL359220.1 | 0.42 | 0.20 | 0.90 | 0.02 |
| AP002840.2 | 0.79 | 0.65 | 0.95 | 0.01 |
| AC100778.2 | 0.64 | 0.40 | 1.00 | 0.05 |
| AC026202.2 | 0.71 | 0.53 | 0.95 | 0.02 |
| AC022400.5 | 0.33 | 0.13 | 0.85 | 0.02 |
| AF111169.3 | 0.42 | 0.21 | 0.86 | 0.02 |
| AL356481.1 | 0.52 | 0.30 | 0.89 | 0.02 |
| AC012085.2 | 1.13 | 1.03 | 1.25 | 0.01 |
| AC008124.1 | 0.72 | 0.53 | 0.98 | 0.04 |
| AC008957.1 | 0.56 | 0.36 | 0.88 | 0.01 |
| AL031667.3 | 1.14 | 1.02 | 1.27 | 0.02 |
| SEPSECS-AS1 | 0.44 | 0.23 | 0.83 | 0.01 |
| EBLN3P | 0.96 | 0.92 | 0.99 | 0.01 |
| AP001178.1 | 1.99 | 1.06 | 3.74 | 0.03 |
| MED4-AS1 | 0.47 | 0.24 | 0.92 | 0.03 |
| AL031666.1 | 0.79 | 0.63 | 1.00 | 0.05 |
| AL512303.1 | 0.47 | 0.22 | 0.99 | 0.05 |
| AC024060.2 | 0.92 | 0.85 | 0.99 | 0.03 |
| AC024075.3 | 0.75 | 0.60 | 0.94 | 0.01 |
| SEMA3F-AS1 | 0.67 | 0.45 | 0.98 | 0.04 |
| AC005884.1 | 0.36 | 0.15 | 0.84 | 0.02 |
| AC022400.6 | 0.70 | 0.51 | 0.96 | 0.03 |
| AP001486.2 | 0.63 | 0.41 | 0.96 | 0.03 |
| AC073517.1 | 0.32 | 0.11 | 0.94 | 0.04 |
| IFNG-AS1 | 0.63 | 0.40 | 0.99 | 0.05 |
| PAN3-AS1 | 0.57 | 0.36 | 0.89 | 0.01 |
| EP300-AS1 | 0.83 | 0.70 | 0.98 | 0.03 |
| SH3BP5-AS1 | 0.85 | 0.76 | 0.96 | 0.01 |
| MIR99AHG | 0.46 | 0.25 | 0.85 | 0.01 |
| IRF1-AS1 | 0.79 | 0.63 | 0.99 | 0.04 |
| TSPOAP1-AS1 | 0.31 | 0.14 | 0.68 | 0.00 |
| AP4B1-AS1 | 0.62 | 0.40 | 0.98 | 0.04 |
