# Supplementary figures and images for: Relationships of N6-Methyladenosine-Related Long Non-Coding RNAs With Tumor Immune Microenvironment and Clinical Prognosis in Lung Adenocarcinoma
Source: Front Genet. 2021 Oct 20;12:714697. doi: 10.3389/fgene.2021.714697 (PMC8585518; doi:10.3389/fgene.2021.714697)

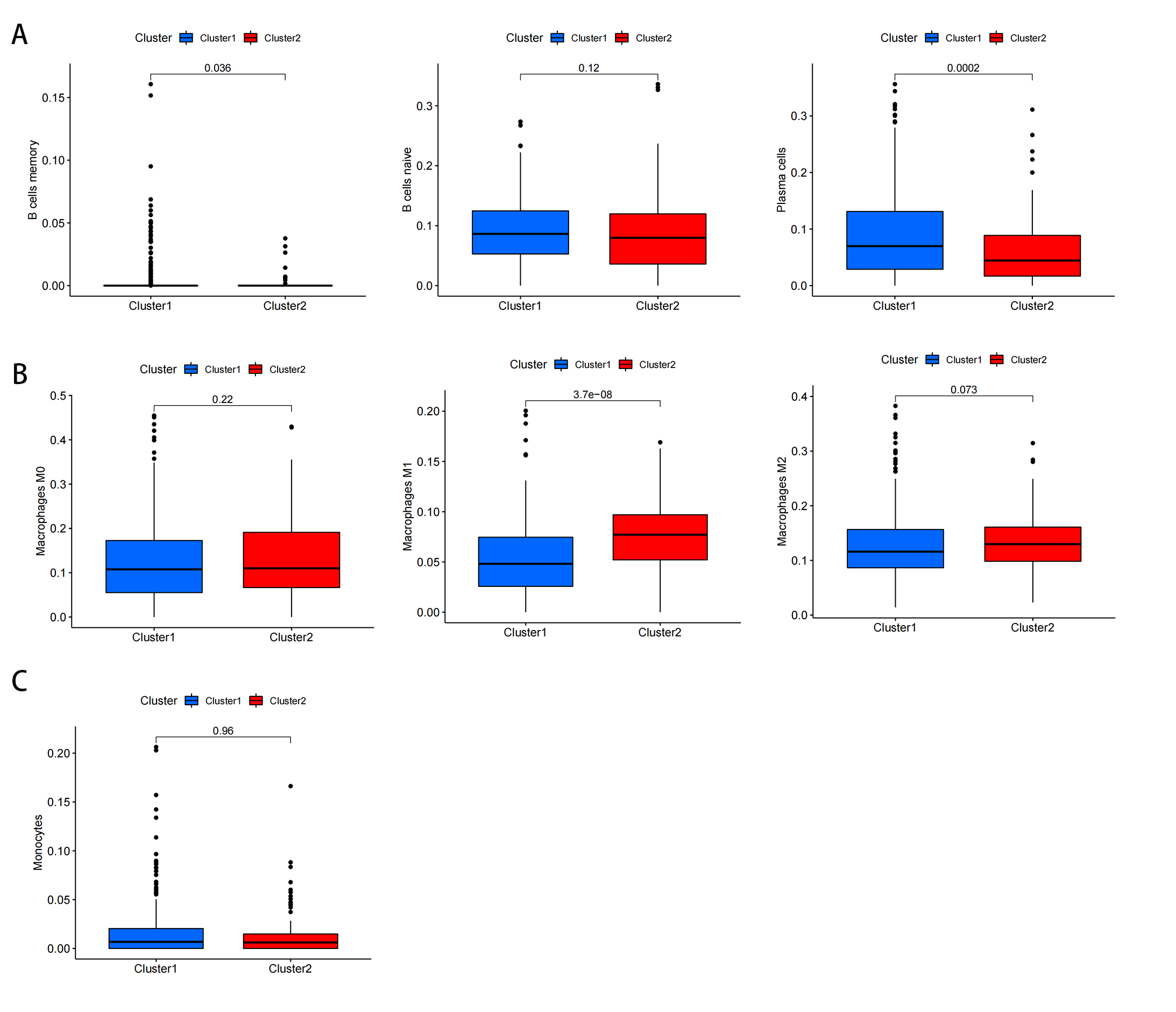

Supplement: Supplementary file 3 [file Image3.TIF]

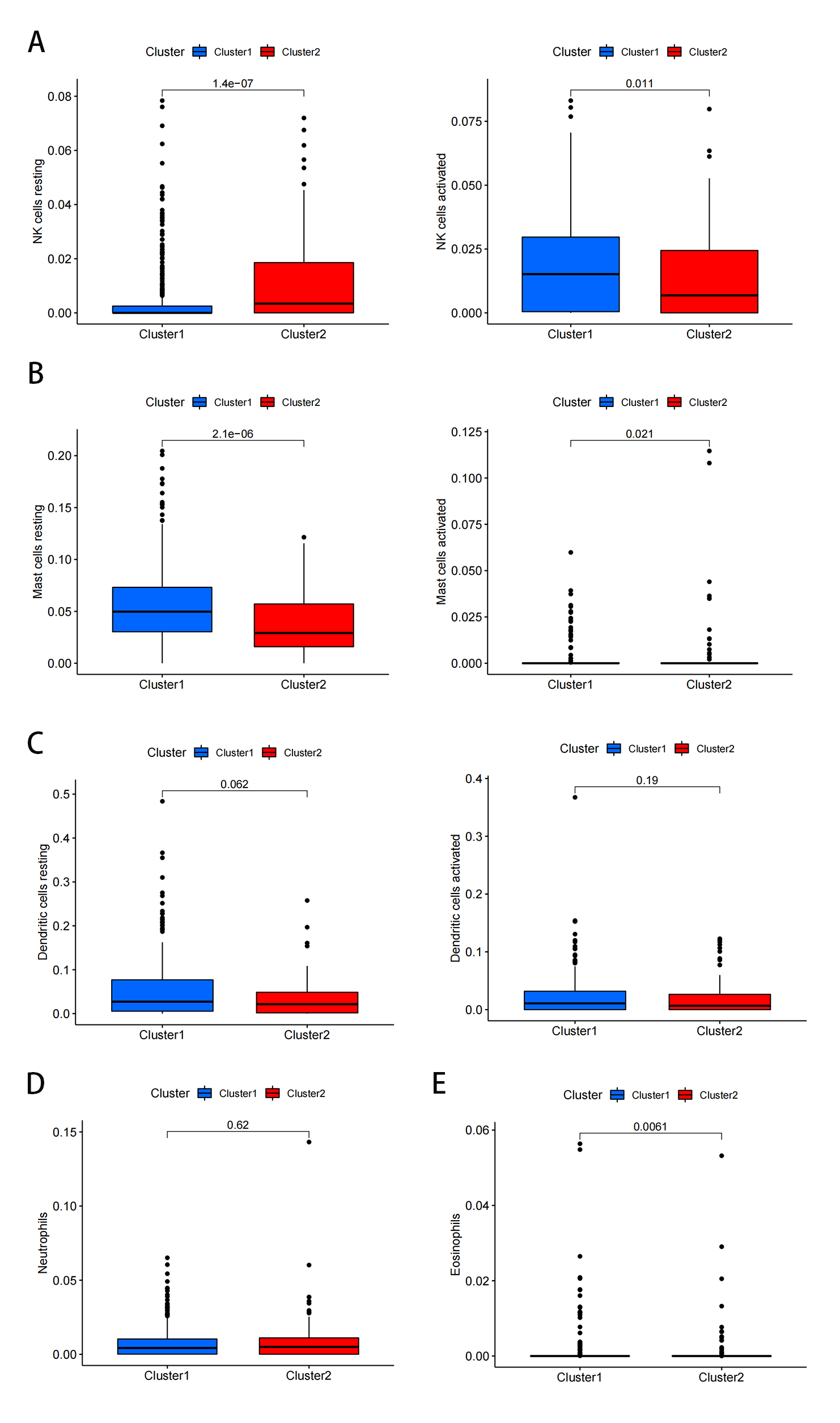

Supplement: Supplementary file 4 [file Image4.TIF]

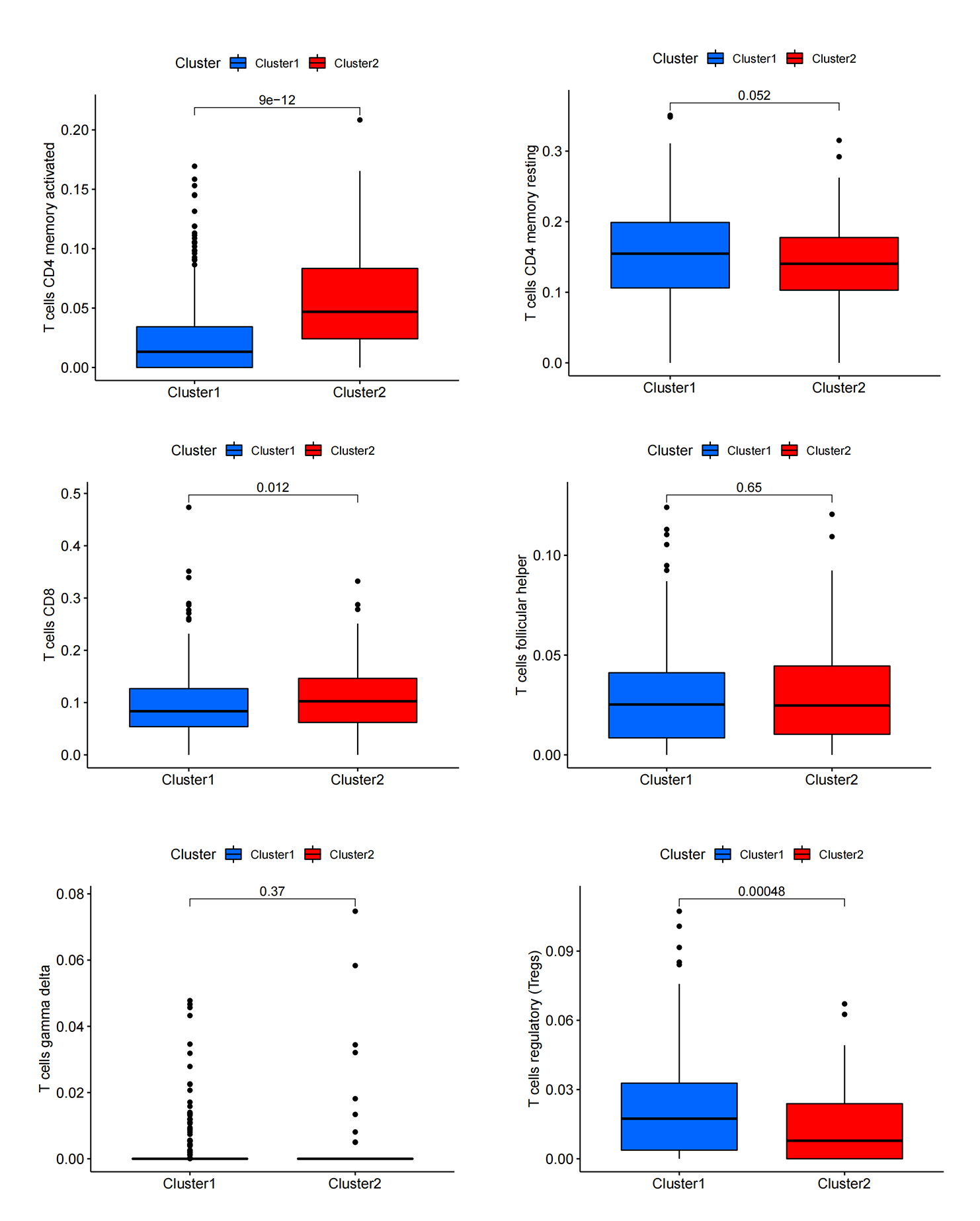

Supplement: Supplementary file 5 [file Image2.TIF]

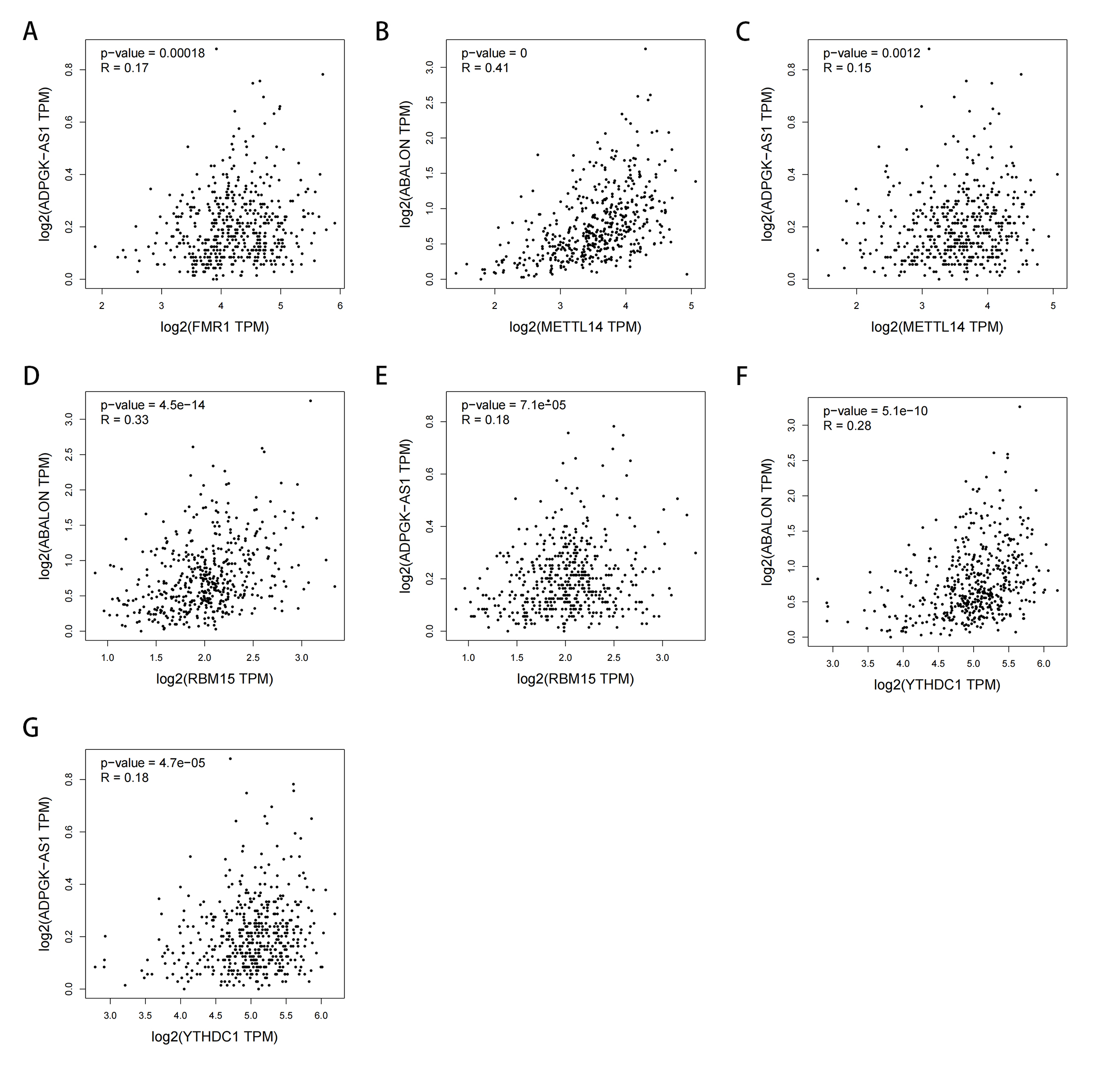

Supplement: Supplementary file 6 [file Image7.JPEG]

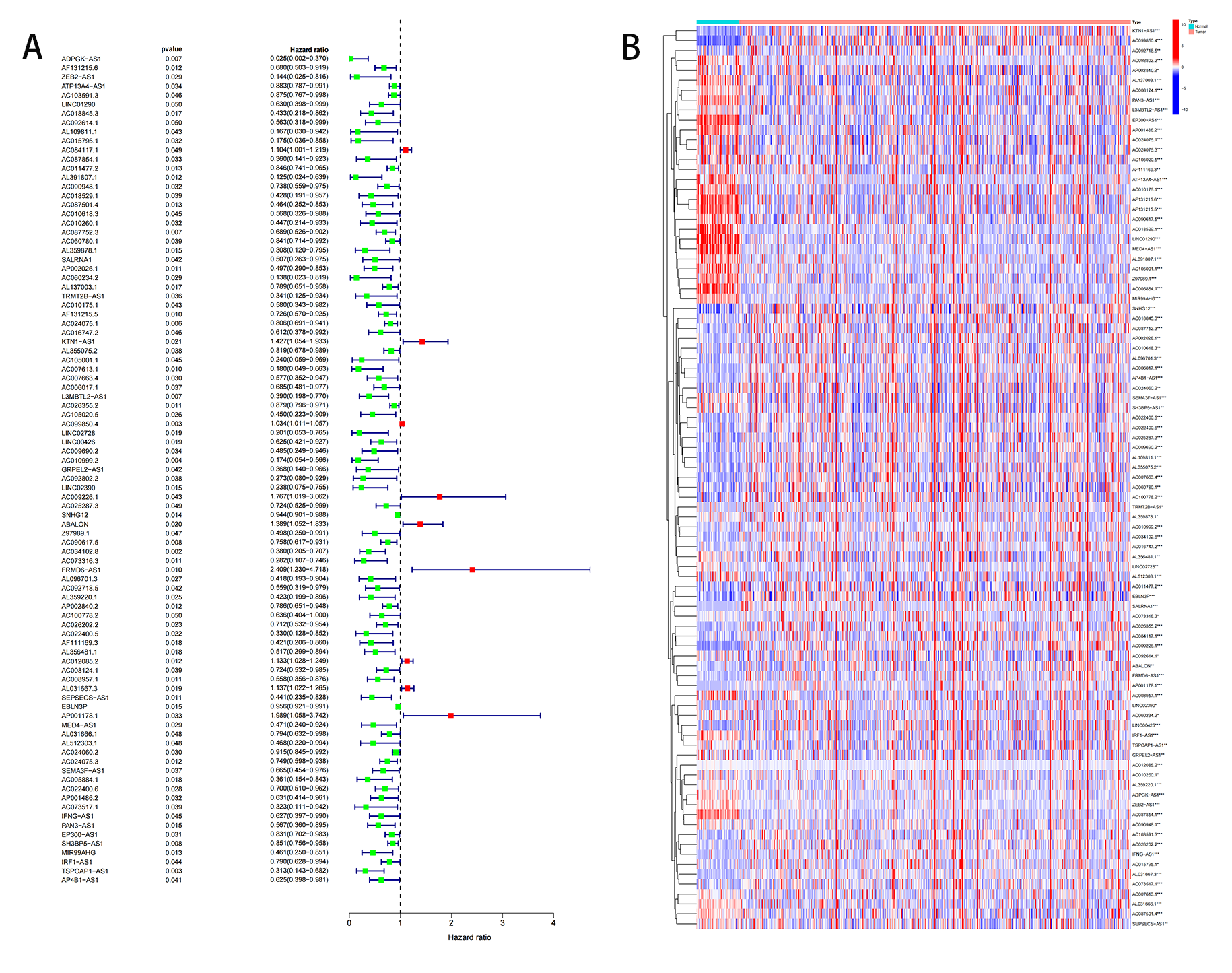

Supplement: Supplementary file 7 [file Image1.TIF]

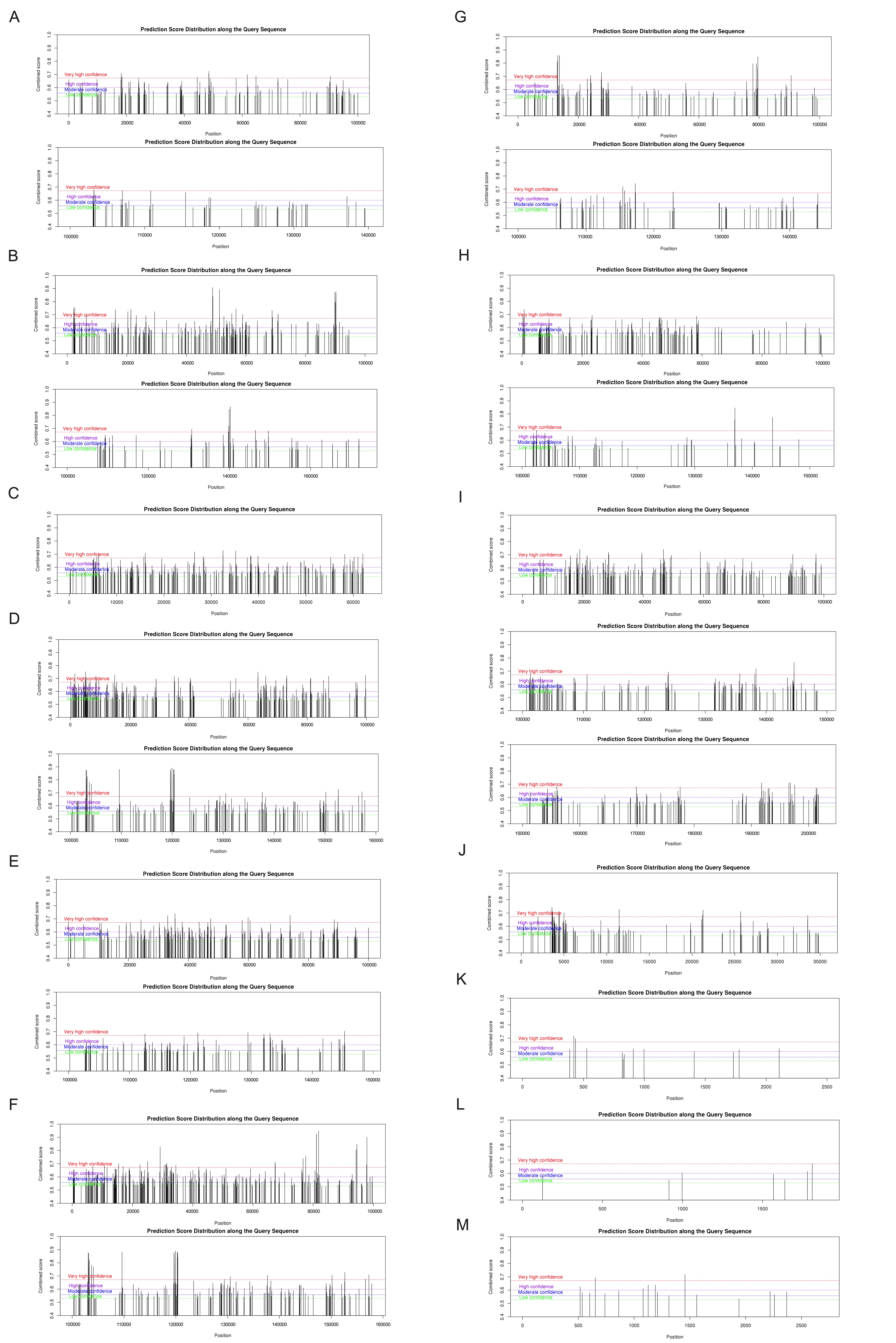

Supplement: Supplementary file 8 [file Image8.JPEG]

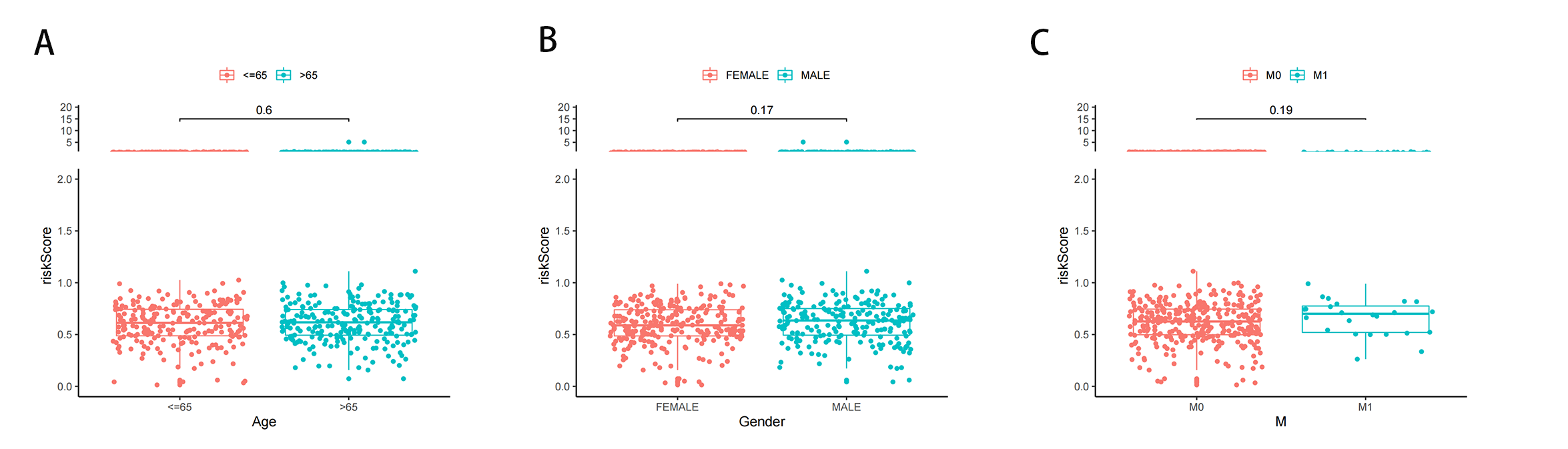

Supplement: Supplementary file 9 [file Image5.TIF]

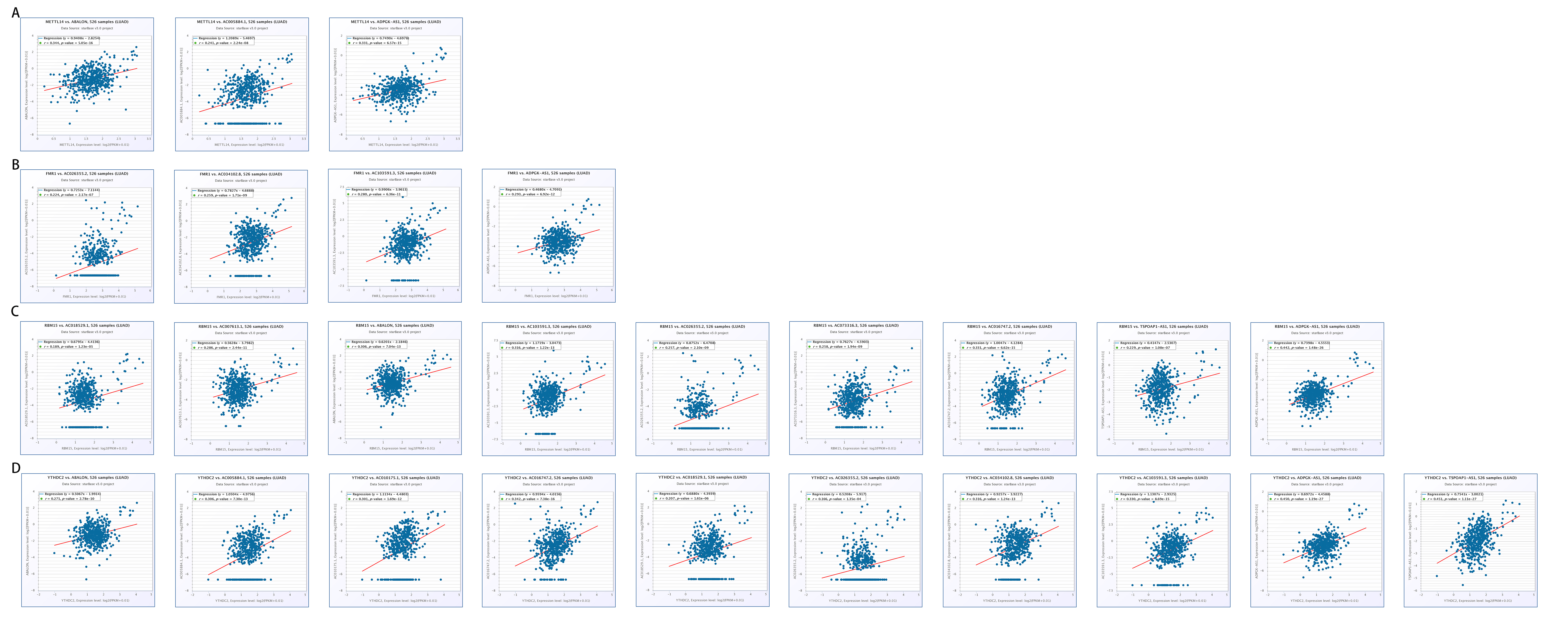

Supplement: Supplementary file 10 [file Image6.JPEG]
